# Supplementary material for: MRI-Based Radiomics Combined with Deep Learning for Distinguishing IDH-Mutant WHO Grade 4 Astrocytomas from IDH-Wild-Type Glioblastomas
Source: Cancers (Basel). 2023 Feb 2;15(3):951. doi: 10.3390/cancers15030951 (PMC9913426; doi:10.3390/cancers15030951)
Supplement: Supplementary file 1 [file cancers-15-00951-s001.zip › cancers-2087681-supplementary.pdf]

Supplementary file

Table S1. Radiomics features' name, set, and family extracted in this study

Set	Type	Name
**Original**	shape	Maximum3DDiameter
**Original**	shape	Maximum2DDiameterSlice
**Original**	shape	Sphericity
**Original**	shape	MinorAxis
**Original**	shape	Elongation
**Original**	shape	SurfaceVolumeRatio
**Original**	shape	Volume
**Original**	shape	MajorAxis
**Original**	shape	SurfaceArea
**Original**	shape	Flatness
**Original**	shape	LeastAxis
**Original**	shape	Maximum2DDiameterColumn
**Original**	shape	Maximum2DDiameterRow
**Original**	gldm	DependenceEntropy
**Original**	gldm	DependenceNonUniformity
**Original**	gldm	GrayLevelNonUniformity
**Original**	gldm	SmallDependenceEmphasis
**Original**	gldm	SmallDependenceHighGrayLevelEmphasis
**Original**	gldm	DependenceNonUniformityNormalized
**Original**	gldm	LargeDependenceEmphasis
**Original**	gldm	LargeDependenceLowGrayLevelEmphasis
**Original**	gldm	DependenceVariance
**Original**	gldm	LargeDependenceHighGrayLevelEmphasis
**Original**	gldm	SmallDependenceLowGrayLevelEmphasis
**Original**	firstorder	InterquartileRange
**Original**	firstorder	Skewness
**Original**	firstorder	Median
**Original**	firstorder	Energy
**Original**	firstorder	RobustMeanAbsoluteDeviation
**Original**	firstorder	MeanAbsoluteDeviation
**Original**	firstorder	TotalEnergy
**Original**	firstorder	Maximum
**Original**	firstorder	RootMeanSquared

**Original**	firstorder	90Percentile
**Original**	firstorder	Minimum
**Original**	firstorder	Range
**Original**	firstorder	Variance
**Original**	firstorder	10Percentile
**Original**	firstorder	Kurtosis
**Original**	firstorder	Mean
**Original**	glrlm	ShortRunLowGrayLevelEmphasis
**Original**	glrlm	RunVariance
**Original**	glrlm	GrayLevelNonUniformity
**Original**	glrlm	LongRunEmphasis
**Original**	glrlm	ShortRunHighGrayLevelEmphasis
**Original**	glrlm	RunLengthNonUniformity
**Original**	glrlm	ShortRunEmphasis
**Original**	glrlm	LongRunHighGrayLevelEmphasis
**Original**	glrlm	RunPercentage
**Original**	glrlm	LongRunLowGrayLevelEmphasis
**Original**	glrlm	RunEntropy
**Original**	glrlm	RunLengthNonUniformityNormalized
**Original**	glszm	LargeAreaEmphasis
**Original**	glszm	SmallAreaHighGrayLevelEmphasis
**Original**	glszm	ZonePercentage
**Original**	glszm	LargeAreaLowGrayLevelEmphasis
**Original**	glszm	LargeAreaHighGrayLevelEmphasis
**Original**	glszm	SmallAreaEmphasis
**Original**	glszm	SmallAreaLowGrayLevelEmphasis

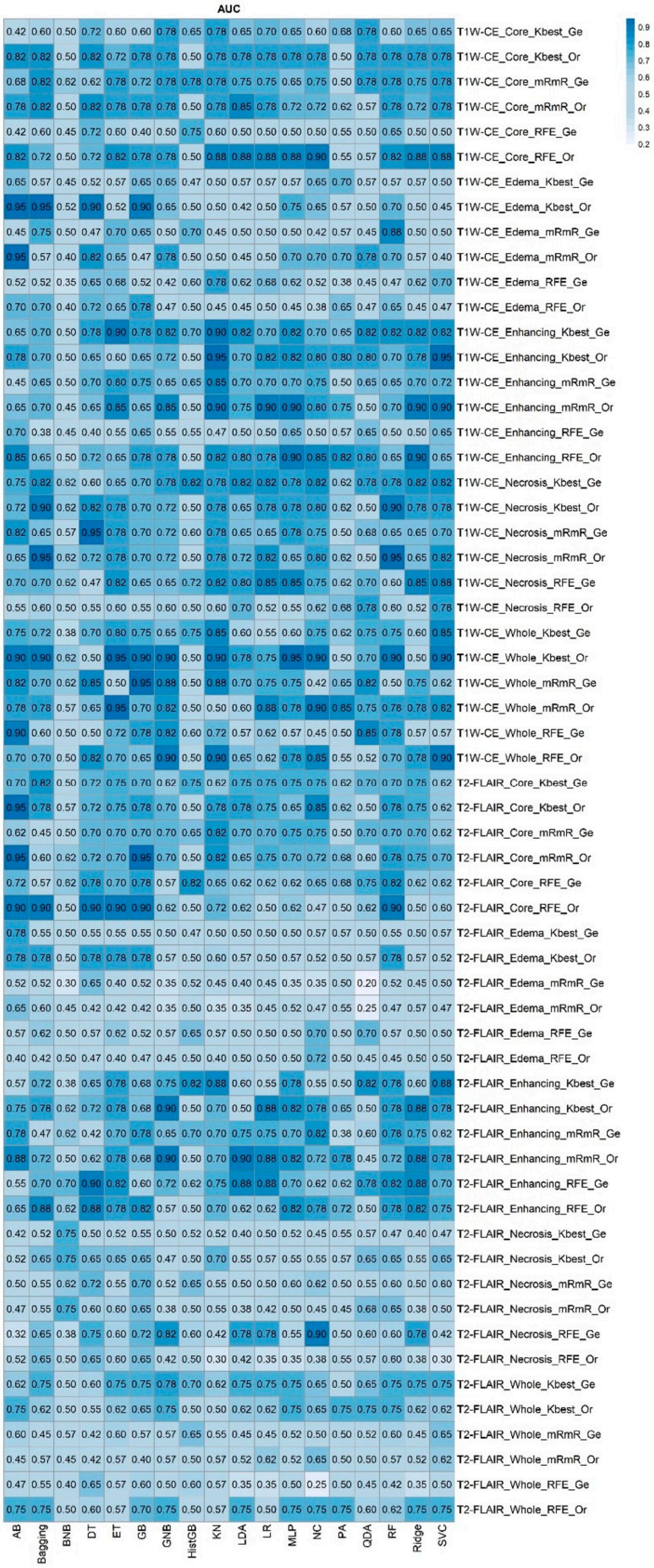

Figure S1. The area under the curve of AUC (predictive power) heatmap for differentiating IDH-mutant from IDH wild-type grade- 4 astrocytomas employing a variety of feature selections and machine learning classifiers applied to distinct neoplasm subregions

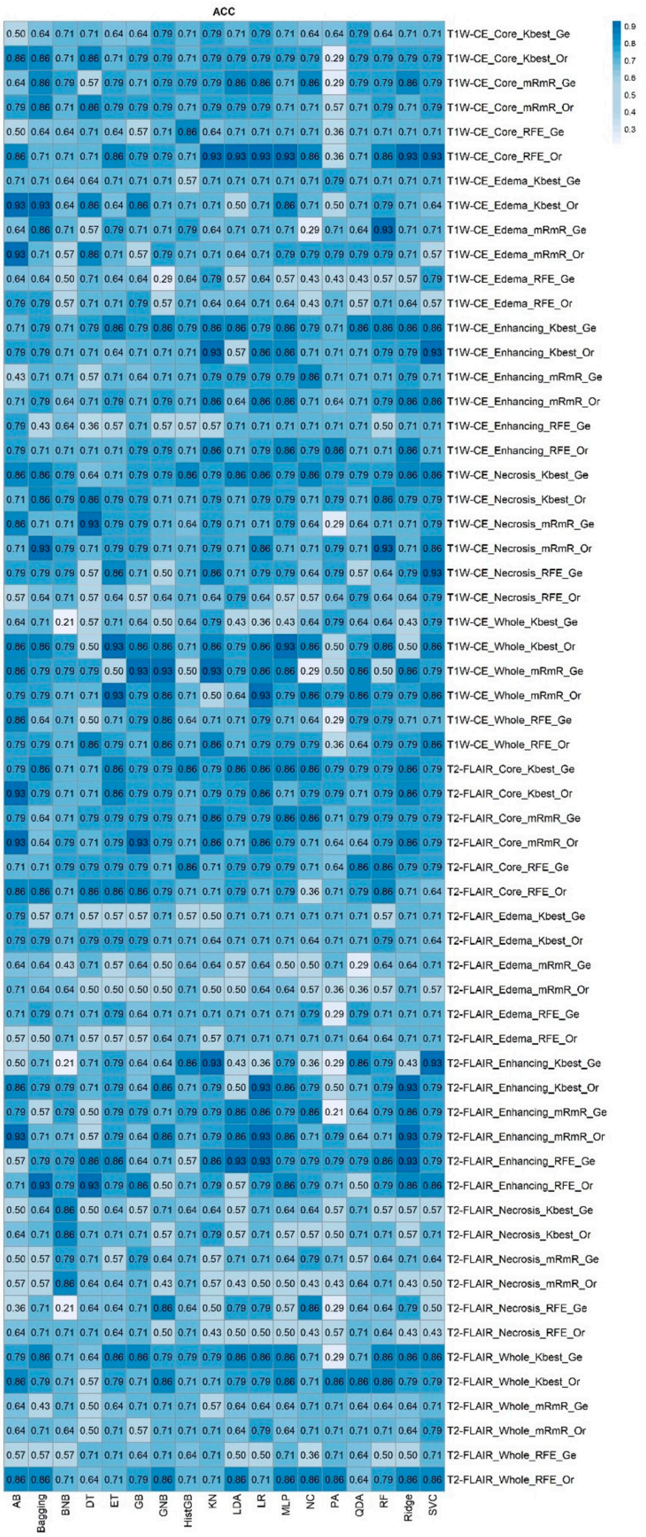

Figure S2. Accuracy (ACC) heatmap for differentiating IDH-mutant from IDH wild-type grade- 4 astrocytomas employing a variety of feature selections and machine learning classifiers applied to distinct neoplasm subregions.

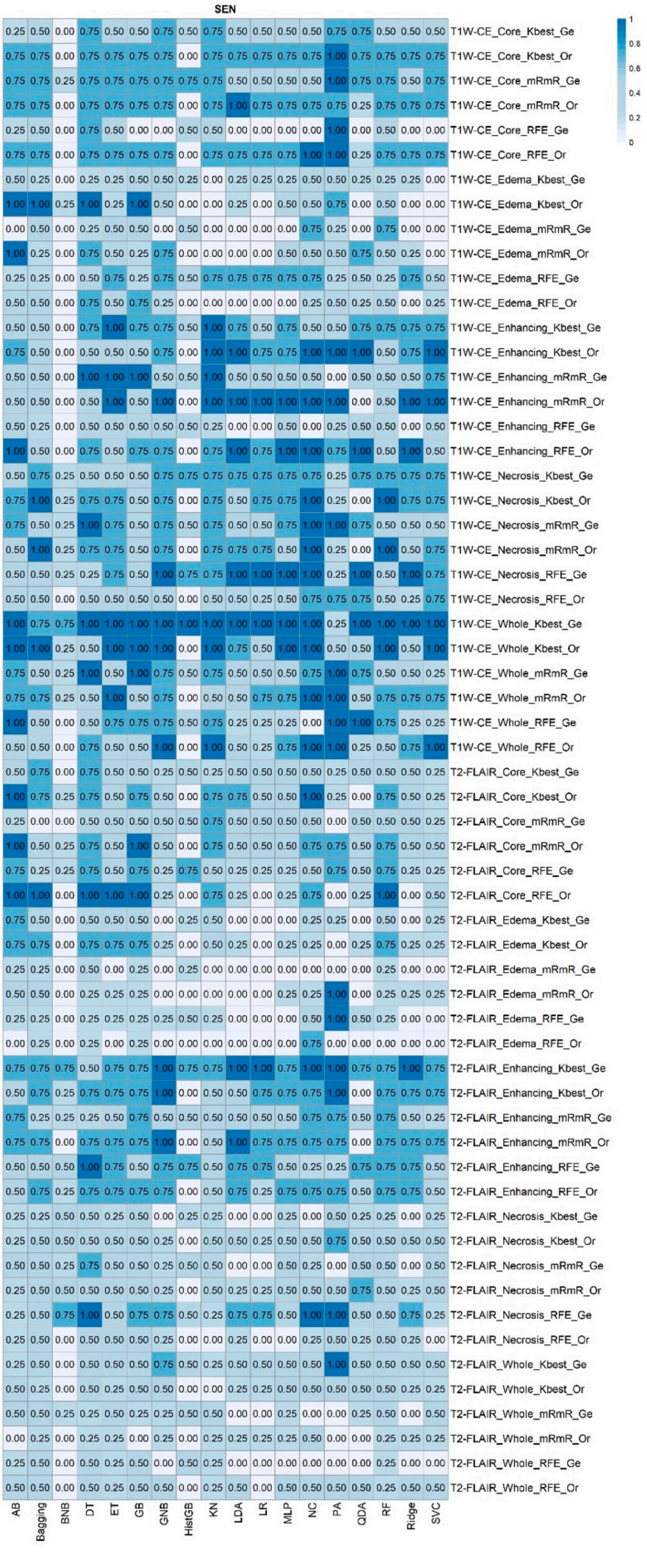

Figure S3. Sensitivity (SEN) heatmap for differentiating IDH-mutant from IDH wild-type grade- 4 astrocytomas employing a variety of feature selections and machine learning classifiers applied to distinct neoplasm.

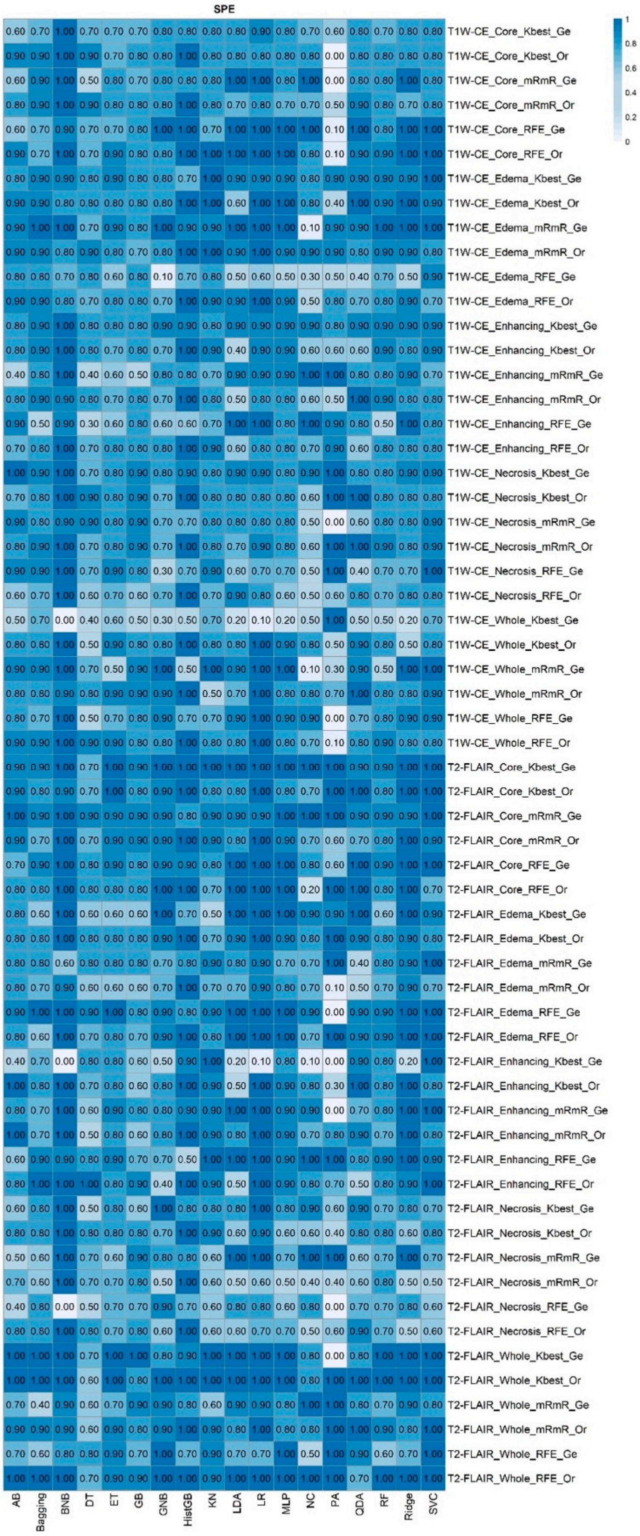

Figure S4. Specificity (SPE) heatmap for differentiating IDH-mutant from IDH wild-type grade- 4 astrocytomas employing a variety of feature selections and machine learning classifiers applied to distinct neoplasm.

Data splitting:

Original data: ['IDH_Mutant_1', 'IDH_Mutant_2', 'IDH_Mutant_3', 'IDH_Mutant_4', 'IDH_Mutant_5', 'IDH_Mutant_6', 'IDH_Mutant_7', 'IDH_Mutant_8', 'IDH_Mutant_9', 'IDH_Mutant_10', 'IDH_Mutant_11', 'IDH_Mutant_12', 'IDH_Mutant_13', 'IDH_Mutant_14', 'IDH_Mutant_15', 'IDH_Mutant_16', 'IDH_Mutant_17', 'IDH_Mutant_18', 'IDH_Mutant_19', 'IDH_Mutant_20', 'IDH_Mutant_21', 'IDH_Mutant_22', 'IDH_Mutant_23', 'IDH_Wild_type_1', 'IDH_Wild_type_2', 'IDH_Wild_type_3', 'IDH_Wild_type_4', 'IDH_Wild_type_5', 'IDH_Wild_type_6', 'IDH_Wild_type_7', 'IDH_Wild_type_8', 'IDH_Wild_type_9', 'IDH_Wild_type_10', 'IDH_Wild_type_11', 'IDH_Wild_type_12', 'IDH_Wild_type_13', 'IDH_Wild_type_14', 'IDH_Wild_type_15', 'IDH_Wild_type_16', 'IDH_Wild_type_17', 'IDH_Wild_type_18', 'IDH_Wild_type_19', 'IDH_Wild_type_20', 'IDH_Wild_type_21', 'IDH_Wild_type_22', 'IDH_Wild_type_23', 'IDH_Wild_type_24', 'IDH_Wild_type_25', 'IDH_Wild_type_26', 'IDH_Wild_type_27', 'IDH_Wild_type_28', 'IDH_Wild_type_29', 'IDH_Wild_type_30', 'IDH_Wild_type_31', 'IDH_Wild_type_32', 'IDH_Wild_type_33', 'IDH_Wild_type_34']

Training set: ['IDH_Mutant_3', 'IDH_Mutant_23', 'IDH_Mutant_19', 'IDH_Wild_type_7', 'IDH_Wild_type_9', 'IDH_Mutant_5', 'IDH_Wild_type_1', 'IDH_Mutant_13', 'IDH_Wild_type_32', 'IDH_Wild_type_17', 'IDH_Wild_type_26', 'IDH_Wild_type_2', 'IDH_Wild_type_34', 'IDH_Wild_type_22', 'IDH_Wild_type_33', 'IDH_Wild_type_30', 'IDH_Wild_type_10', 'IDH_Mutant_14', 'IDH_Wild_type_16', 'IDH_Mutant_10', 'IDH_Wild_type_29', 'IDH_Wild_type_18', 'IDH_Mutant_1', 'IDH_Mutant_20', 'IDH_Wild_type_15', 'IDH_Wild_type_23', 'IDH_Wild_type_8', 'IDH_Mutant_21', 'IDH_Wild_type_6', 'IDH_Wild_type_4', 'IDH_Mutant_18', 'IDH_Mutant_15', 'IDH_Wild_type_12', 'IDH_Mutant_11', 'IDH_Mutant_16', 'IDH_Mutant_8', 'IDH_Wild_type_11', 'IDH_Wild_type_28', 'IDH_Mutant_2', 'IDH_Wild_type_13', 'IDH_Mutant_6', 'IDH_Wild_type_21', 'IDH_Wild_type_31', 'IDH_Wild_type_20', 'IDH_Mutant_9', 'IDH_Mutant_22']

Testing set: ['IDH_Mutant_4', 'IDH_Mutant_7', 'IDH_Mutant_12', 'IDH_Mutant_17', 'IDH_Wild_type_3', 'IDH_Wild_type_5', 'IDH_Wild_type_14', 'IDH_Wild_type_19', 'IDH_Wild_type_24', 'IDH_Wild_type_25', 'IDH_Wild_type_27']

Table S2. The complete result of multi-machine learning algorithms, feature selection, and multi-segmentation approaches in discriminating IDH-mutant grade-4 astrocytomas from IDH wild-type GBMs of generated data with 80:20 training:testing set.

Approach	ML	AUC	ACC	SEN	SPE	PR_AUC
T1w_CE_Core_Kbest	GNB	0.92857143	0.91666667	1	0.85714286	0.91666667
T1w_CE_Core_RFE	PA	0.92857143	0.91666667	1	0.85714286	0.91666667
T1w_CE_Enhancing_mRmR	HGB	0.92857143	0.91666667	1	0.85714286	0.91666667
T2w_Flair_Core_RFE	KN	0.92857143	0.91666667	1	0.85714286	0.91666667
T2w_Flair_Enhancing_Kbest	Bagging	0.92857143	0.91666667	1	0.85714286	0.91666667
T2w_Flair_Enhancing_Kbest	DT	0.92857143	0.91666667	1	0.85714286	0.91666667
T2w_Flair_Enhancing_Kbest	KN	0.92857143	0.91666667	1	0.85714286	0.91666667
T1w_CE_Core_Kbest	Bagging	0.9	0.91666667	0.8	1	0.94166667
T1w_CE_Core_Kbest	GB	0.9	0.91666667	0.8	1	0.94166667
T1w_CE_Core_Kbest	HGB	0.9	0.91666667	0.8	1	0.94166667
T1w_CE_Core_Kbest	RF	0.9	0.91666667	0.8	1	0.94166667

T1w_CE_Core_RFE	RF	0.9	0.91666667	0.8	1	0.94166667
T1w_CE_Edema_Kbest	GNB	0.9	0.91666667	0.8	1	0.94166667
T1w_CE_Edema_Kbest	MLPC	0.9	0.91666667	0.8	1	0.94166667
T1w_CE_Edema_Kbest	NC	0.9	0.91666667	0.8	1	0.94166667
T1w_CE_Edema_Kbest	PA	0.9	0.91666667	0.8	1	0.94166667
T1w_CE_Edema_Kbest	QDA	0.9	0.91666667	0.8	1	0.94166667
T1w_CE_Edema_Kbest	SVC	0.9	0.91666667	0.8	1	0.94166667
T1w_CE_Edema_RFE	NC	0.9	0.91666667	0.8	1	0.94166667
T1w_CE_Enhancing_Kbest	QDA	0.9	0.91666667	0.8	1	0.94166667
T1w_CE_Enhancing_mRmR	NC	0.9	0.91666667	0.8	1	0.94166667
T1w_CE_Enhancing_mRmR	SVC	0.9	0.91666667	0.8	1	0.94166667
T1w_CE_Enhancing_RFE	ET	0.9	0.91666667	0.8	1	0.94166667
T1w_CE_Enhancing_RFE	GB	0.9	0.91666667	0.8	1	0.94166667
T1w_CE_Enhancing_RFE	KN	0.9	0.91666667	0.8	1	0.94166667
T1w_CE_Enhancing_RFE	RF	0.9	0.91666667	0.8	1	0.94166667
T1w_CE_Enhancing_RFE	SVC	0.9	0.91666667	0.8	1	0.94166667
T1w_CE_Whole_Kbest	ET	0.9	0.91666667	0.8	1	0.94166667
T1w_CE_Whole_Kbest	GB	0.9	0.91666667	0.8	1	0.94166667
T1w_CE_Whole_Kbest	HGB	0.9	0.91666667	0.8	1	0.94166667
T1w_CE_Whole_Kbest	SVC	0.9	0.91666667	0.8	1	0.94166667
T2w_Flair_Whole_Kbest	AB	0.9	0.91666667	0.8	1	0.94166667
T1w_CE_Core_Kbest	LDA	0.85714286	0.83333333	1	0.71428571	0.85714286
T1w_CE_Core_Kbest	LR	0.85714286	0.83333333	1	0.71428571	0.85714286
T1w_CE_Core_Kbest	MLPC	0.85714286	0.83333333	1	0.71428571	0.85714286
T1w_CE_Core_Kbest	NC	0.85714286	0.83333333	1	0.71428571	0.85714286
T1w_CE_Core_Kbest	Ridge	0.85714286	0.83333333	1	0.71428571	0.85714286
T1w_CE_Enhancing_Kbest	PA	0.85714286	0.83333333	1	0.71428571	0.85714286
T1w_CE_Enhancing_mRmR	AB	0.85714286	0.83333333	1	0.71428571	0.85714286
T1w_CE_Necrosis_Kbest	NC	0.85714286	0.83333333	1	0.71428571	0.85714286
T2w_Flair_Core_RFE	HGB	0.85714286	0.83333333	1	0.71428571	0.85714286
T2w_Flair_Enhancing_Kbest	GB	0.85714286	0.83333333	1	0.71428571	0.85714286
T1w_CE_Core_Kbest	AB	0.82857143	0.83333333	0.8	0.85714286	0.84166667
T1w_CE_Core_mRmR	GB	0.82857143	0.83333333	0.8	0.85714286	0.84166667
T1w_CE_Core_mRmR	SVC	0.82857143	0.83333333	0.8	0.85714286	0.84166667
T1w_CE_Enhancing_mRmR	PA	0.82857143	0.83333333	0.8	0.85714286	0.84166667
T1w_CE_Enhancing_RFE	Bagging	0.82857143	0.83333333	0.8	0.85714286	0.84166667
T1w_CE_Enhancing_RFE	DT	0.82857143	0.83333333	0.8	0.85714286	0.84166667
T1w_CE_Enhancing_RFE	HGB	0.82857143	0.83333333	0.8	0.85714286	0.84166667
T1w_CE_Whole_Kbest	KN	0.82857143	0.83333333	0.8	0.85714286	0.84166667
T1w_CE_Whole_Kbest	NC	0.82857143	0.83333333	0.8	0.85714286	0.84166667

T1w_CE_Whole_RFE	RF	0.82857143	0.83333333	0.8	0.85714286	0.84166667
T2w_Flair_Core_Kbest	NC	0.82857143	0.83333333	0.8	0.85714286	0.84166667
T2w_Flair_Core_mRmR	Bagging	0.82857143	0.83333333	0.8	0.85714286	0.84166667
T2w_Flair_Core_mRmR	ET	0.82857143	0.83333333	0.8	0.85714286	0.84166667
T2w_Flair_Core_mRmR	GB	0.82857143	0.83333333	0.8	0.85714286	0.84166667
T2w_Flair_Core_mRmR	HGB	0.82857143	0.83333333	0.8	0.85714286	0.84166667
T2w_Flair_Core_mRmR	KN	0.82857143	0.83333333	0.8	0.85714286	0.84166667
T2w_Flair_Core_mRmR	QDA	0.82857143	0.83333333	0.8	0.85714286	0.84166667
T2w_Flair_Core_mRmR	RF	0.82857143	0.83333333	0.8	0.85714286	0.84166667
T2w_Flair_Core_mRmR	SVC	0.82857143	0.83333333	0.8	0.85714286	0.84166667
T2w_Flair_Core_RFE	GNB	0.82857143	0.83333333	0.8	0.85714286	0.84166667
T2w_Flair_Core_RFE	LDA	0.82857143	0.83333333	0.8	0.85714286	0.84166667
T2w_Flair_Core_RFE	MLPC	0.82857143	0.83333333	0.8	0.85714286	0.84166667
T2w_Flair_Core_RFE	QDA	0.82857143	0.83333333	0.8	0.85714286	0.84166667
T2w_Flair_Core_RFE	SVC	0.82857143	0.83333333	0.8	0.85714286	0.84166667
T2w_Flair_Enhancing_Kbest	ET	0.82857143	0.83333333	0.8	0.85714286	0.84166667
T2w_Flair_Enhancing_Kbest	HGB	0.82857143	0.83333333	0.8	0.85714286	0.84166667
T2w_Flair_Enhancing_Kbest	RF	0.82857143	0.83333333	0.8	0.85714286	0.84166667
T2w_Flair_Enhancing_Kbest	HGB	0.82857143	0.83333333	0.8	0.85714286	0.84166667
T2w_Flair_Enhancing_Kbest	NC	0.82857143	0.83333333	0.8	0.85714286	0.84166667
T2w_Flair_Enhancing_mRmR	ET	0.82857143	0.83333333	0.8	0.85714286	0.84166667
T2w_Flair_Enhancing_mRmR	RF	0.82857143	0.83333333	0.8	0.85714286	0.84166667
T2w_Flair_Enhancing_mRmR	AB	0.82857143	0.83333333	0.8	0.85714286	0.84166667
T2w_Flair_Enhancing_mRmR	SVC	0.82857143	0.83333333	0.8	0.85714286	0.84166667
T2w_Flair_Enhancing_RFE	NC	0.82857143	0.83333333	0.8	0.85714286	0.84166667
T2w_Flair_Necrosis_Kbest	AB	0.82857143	0.83333333	0.8	0.85714286	0.84166667
T1w_CE_Core_Kbest	DT	0.8	0.83333333	0.6	1	0.88333333
T1w_CE_Core_RFE	AB	0.8	0.83333333	0.6	1	0.88333333
T1w_CE_Core_RFE	ET	0.8	0.83333333	0.6	1	0.88333333
T1w_CE_Core_RFE	HGB	0.8	0.83333333	0.6	1	0.88333333
T1w_CE_Core_RFE	KN	0.8	0.83333333	0.6	1	0.88333333
T1w_CE_Edema_mRmR	QDA	0.8	0.83333333	0.6	1	0.88333333
T1w_CE_Enhancing_Kbest	ET	0.8	0.83333333	0.6	1	0.88333333
T1w_CE_Enhancing_Kbest	GNB	0.8	0.83333333	0.6	1	0.88333333
T1w_CE_Enhancing_Kbest	GB	0.8	0.83333333	0.6	1	0.88333333
T1w_CE_Enhancing_Kbest	HGB	0.8	0.83333333	0.6	1	0.88333333
T1w_CE_Enhancing_Kbest	RF	0.8	0.83333333	0.6	1	0.88333333
T1w_CE_Enhancing_mRmR	DT	0.8	0.83333333	0.6	1	0.88333333
T1w_CE_Enhancing_mRmR	ET	0.8	0.83333333	0.6	1	0.88333333
T1w_CE_Enhancing_mRmR	RF	0.8	0.83333333	0.6	1	0.88333333

T1w_CE_Enhancing_RFE	MLPC	0.8	0.83333333	0.6	1	0.88333333
T1w_CE_Enhancing_RFE	QDA	0.8	0.83333333	0.6	1	0.88333333
T1w_CE_Whole_Kbest	DT	0.8	0.83333333	0.6	1	0.88333333
T1w_CE_Whole_Kbest	MLPC	0.8	0.83333333	0.6	1	0.88333333
T1w_CE_Whole_Kbest	RF	0.8	0.83333333	0.6	1	0.88333333
T1w_CE_Whole_RFE	DT	0.8	0.83333333	0.6	1	0.88333333
T2w_Flair_Core_mRmR	AB	0.8	0.83333333	0.6	1	0.88333333
T2w_Flair_Enhancing_Kbest	SVC	0.8	0.83333333	0.6	1	0.88333333
T2w_Flair_Enhancing_RFE	GB	0.8	0.83333333	0.6	1	0.88333333
T2w_Flair_Enhancing_RFE	KN	0.8	0.83333333	0.6	1	0.88333333
T2w_Flair_Enhancing_RFE	RF	0.8	0.83333333	0.6	1	0.88333333
T2w_Flair_Whole_Kbest	Bagging	0.8	0.83333333	0.6	1	0.88333333
T2w_Flair_Whole_Kbest	ET	0.8	0.83333333	0.6	1	0.88333333
T2w_Flair_Whole_Kbest	GB	0.8	0.83333333	0.6	1	0.88333333
T2w_Flair_Whole_Kbest	KN	0.8	0.83333333	0.6	1	0.88333333
T2w_Flair_Whole_Kbest	RF	0.8	0.83333333	0.6	1	0.88333333
T2w_Flair_Whole_mRmR	ET	0.8	0.83333333	0.6	1	0.88333333
T2w_Flair_Whole_mRmR	KN	0.8	0.83333333	0.6	1	0.88333333
T2w_Flair_Whole_mRmR	RF	0.8	0.83333333	0.6	1	0.88333333
T2w_Flair_Whole_RFE	GB	0.8	0.83333333	0.6	1	0.88333333
T1w_CE_Edema_Kbest	AB	0.78571429	0.75	1	0.57142857	0.8125
T1w_CE_Enhancing_RFE	AB	0.78571429	0.75	1	0.57142857	0.8125
T1w_CE_Core_mRmR	MLPC	0.75714286	0.75	0.8	0.71428571	0.775
T1w_CE_Core_mRmR	QDA	0.75714286	0.75	0.8	0.71428571	0.775
T1w_CE_Enhancing_mRmR	GB	0.75714286	0.75	0.8	0.71428571	0.775
T1w_CE_Necrosis_Kbest	AB	0.75714286	0.75	0.8	0.71428571	0.775
T1w_CE_Necrosis_RFE	SVC	0.75714286	0.75	0.8	0.71428571	0.775
T1w_CE_Whole_RFE	ET	0.75714286	0.75	0.8	0.71428571	0.775
T1w_CE_Whole_RFE	HGB	0.75714286	0.75	0.8	0.71428571	0.775
T2w_Flair_Core_RFE	AB	0.75714286	0.75	0.8	0.71428571	0.775
T2w_Flair_Enhancing_mRmR	Bagging	0.75714286	0.75	0.8	0.71428571	0.775
T2w_Flair_Enhancing_mRmR	GB	0.75714286	0.75	0.8	0.71428571	0.775
T1w_CE_Core_mRmR	Bagging	0.72857143	0.75	0.6	0.85714286	0.75833333
T1w_CE_Core_mRmR	GNB	0.72857143	0.75	0.6	0.85714286	0.75833333
T1w_CE_Necrosis_Kbest	SVC	0.72857143	0.75	0.6	0.85714286	0.75833333
T1w_CE_Necrosis_mRmR	KN	0.72857143	0.75	0.6	0.85714286	0.75833333
T1w_CE_Necrosis_mRmR	LDA	0.72857143	0.75	0.6	0.85714286	0.75833333
T1w_CE_Necrosis_mRmR	LR	0.72857143	0.75	0.6	0.85714286	0.75833333
T1w_CE_Necrosis_mRmR	Ridge	0.72857143	0.75	0.6	0.85714286	0.75833333
T1w_CE_Necrosis_mRmR	SVC	0.72857143	0.75	0.6	0.85714286	0.75833333

T1w_CE_Necrosis_RFE	MLPC	0.72857143	0.75	0.6	0.85714286	0.75833333
T1w_CE_Whole_Kbest	AB	0.72857143	0.75	0.6	0.85714286	0.75833333
T1w_CE_Whole_Kbest	Bagging	0.72857143	0.75	0.6	0.85714286	0.75833333
T1w_CE_Whole_Kbest	PA	0.72857143	0.75	0.6	0.85714286	0.75833333
T1w_CE_Whole_mRmR	DT	0.72857143	0.75	0.6	0.85714286	0.75833333
T1w_CE_Whole_mRmR	RF	0.72857143	0.75	0.6	0.85714286	0.75833333
T1w_CE_Whole_RFE	Bagging	0.72857143	0.75	0.6	0.85714286	0.75833333
T1w_CE_Whole_RFE	GB	0.72857143	0.75	0.6	0.85714286	0.75833333
T1w_CE_Whole_RFE	KN	0.72857143	0.75	0.6	0.85714286	0.75833333
T2w_Flair_Core_mRmR	MLPC	0.72857143	0.75	0.6	0.85714286	0.75833333
T2w_Flair_Core_RFE	Ridge	0.72857143	0.75	0.6	0.85714286	0.75833333
T2w_Flair_Edema_Kbest	Bagging	0.72857143	0.75	0.6	0.85714286	0.75833333
T2w_Flair_Edema_RFE	AB	0.72857143	0.75	0.6	0.85714286	0.75833333
T2w_Flair_Edema_RFE	DT	0.72857143	0.75	0.6	0.85714286	0.75833333
T2w_Flair_Enhancing_Kbest	MLPC	0.72857143	0.75	0.6	0.85714286	0.75833333
T2w_Flair_Enhancing_mRmR	MLPC	0.72857143	0.75	0.6	0.85714286	0.75833333
T2w_Flair_Enhancing_mRmR	SVC	0.72857143	0.75	0.6	0.85714286	0.75833333
T2w_Flair_Enhancing_mRmR	KN	0.72857143	0.75	0.6	0.85714286	0.75833333
T2w_Flair_Enhancing_RFE	ET	0.72857143	0.75	0.6	0.85714286	0.75833333
T2w_Flair_Enhancing_RFE	HGB	0.72857143	0.75	0.6	0.85714286	0.75833333
T2w_Flair_Enhancing_RFE	RF	0.72857143	0.75	0.6	0.85714286	0.75833333
T2w_Flair_Whole_Kbest	HGB	0.72857143	0.75	0.6	0.85714286	0.75833333
T2w_Flair_Whole_mRmR	DT	0.72857143	0.75	0.6	0.85714286	0.75833333
T2w_Flair_Whole_RFE	Bagging	0.72857143	0.75	0.6	0.85714286	0.75833333
T2w_Flair_Whole_RFE	ET	0.72857143	0.75	0.6	0.85714286	0.75833333
T2w_Flair_Whole_RFE	GNB	0.72857143	0.75	0.6	0.85714286	0.75833333
T2w_Flair_Whole_RFE	HGB	0.72857143	0.75	0.6	0.85714286	0.75833333
T2w_Flair_Whole_RFE	MLPC	0.72857143	0.75	0.6	0.85714286	0.75833333
T2w_Flair_Whole_RFE	RF	0.72857143	0.75	0.6	0.85714286	0.75833333
T2w_Flair_Whole_RFE	SVC	0.72857143	0.75	0.6	0.85714286	0.75833333
T1w_CE_Core_mRmR	HGB	0.71428571	0.66666667	1	0.42857143	0.77777778
T2w_Flair_Whole_Kbest	DT	0.71428571	0.66666667	1	0.42857143	0.77777778
T1w_CE_Core_mRmR	LR	0.7	0.75	0.4	1	0.825
T1w_CE_Core_mRmR	Ridge	0.7	0.75	0.4	1	0.825
T1w_CE_Core_RFE	Bagging	0.7	0.75	0.4	1	0.825
T1w_CE_Core_RFE	DT	0.7	0.75	0.4	1	0.825
T1w_CE_Core_RFE	GNB	0.7	0.75	0.4	1	0.825
T1w_CE_Core_RFE	GB	0.7	0.75	0.4	1	0.825
T1w_CE_Core_RFE	QDA	0.7	0.75	0.4	1	0.825
T1w_CE_Core_RFE	SVC	0.7	0.75	0.4	1	0.825

T1w_CE_Edema_Kbest	LDA	0.7	0.75	0.4	1	0.825
T1w_CE_Edema_Kbest	LR	0.7	0.75	0.4	1	0.825
T1w_CE_Edema_Kbest	Ridge	0.7	0.75	0.4	1	0.825
T1w_CE_Edema_mRmR	Bagging	0.7	0.75	0.4	1	0.825
T1w_CE_Edema_mRmR	ET	0.7	0.75	0.4	1	0.825
T1w_CE_Edema_mRmR	KN	0.7	0.75	0.4	1	0.825
T1w_CE_Edema_mRmR	MLPC	0.7	0.75	0.4	1	0.825
T1w_CE_Edema_mRmR	SVC	0.7	0.75	0.4	1	0.825
T1w_CE_Enhancing_Kbest	Bagging	0.7	0.75	0.4	1	0.825
T1w_CE_Enhancing_Kbest	DT	0.7	0.75	0.4	1	0.825
T1w_CE_Enhancing_Kbest	KN	0.7	0.75	0.4	1	0.825
T1w_CE_Whole_Kbest	GNB	0.7	0.75	0.4	1	0.825
T1w_CE_Whole_Kbest	LDA	0.7	0.75	0.4	1	0.825
T1w_CE_Whole_Kbest	LR	0.7	0.75	0.4	1	0.825
T1w_CE_Whole_Kbest	QDA	0.7	0.75	0.4	1	0.825
T1w_CE_Whole_Kbest	Ridge	0.7	0.75	0.4	1	0.825
T1w_CE_Whole_mRmR	LDA	0.7	0.75	0.4	1	0.825
T1w_CE_Whole_mRmR	MLPC	0.7	0.75	0.4	1	0.825
T1w_CE_Whole_mRmR	NC	0.7	0.75	0.4	1	0.825
T1w_CE_Whole_mRmR	QDA	0.7	0.75	0.4	1	0.825
T1w_CE_Whole_mRmR	Ridge	0.7	0.75	0.4	1	0.825
T1w_CE_Whole_mRmR	SVC	0.7	0.75	0.4	1	0.825
T1w_CE_Whole_RFE	GNB	0.7	0.75	0.4	1	0.825
T1w_CE_Whole_RFE	MLPC	0.7	0.75	0.4	1	0.825
T1w_CE_Whole_RFE	QDA	0.7	0.75	0.4	1	0.825
T1w_CE_Whole_RFE	SVC	0.7	0.75	0.4	1	0.825
T2w_Flair_Core_mRmR	LDA	0.7	0.75	0.4	1	0.825
T2w_Flair_Core_mRmR	Ridge	0.7	0.75	0.4	1	0.825
T2w_Flair_Edema_Kbest	GNB	0.7	0.75	0.4	1	0.825
T2w_Flair_Edema_Kbest	HGB	0.7	0.75	0.4	1	0.825
T2w_Flair_Edema_mRmR	ET	0.7	0.75	0.4	1	0.825
T2w_Flair_Edema_mRmR	GNB	0.7	0.75	0.4	1	0.825
T2w_Flair_Edema_mRmR	HGB	0.7	0.75	0.4	1	0.825
T2w_Flair_Edema_mRmR	MLPC	0.7	0.75	0.4	1	0.825
T2w_Flair_Edema_mRmR	RF	0.7	0.75	0.4	1	0.825
T2w_Flair_Edema_RFE	ET	0.7	0.75	0.4	1	0.825
T2w_Flair_Edema_RFE	GB	0.7	0.75	0.4	1	0.825
T2w_Flair_Edema_RFE	HGB	0.7	0.75	0.4	1	0.825
T2w_Flair_Enhancing_RFE	SVC	0.7	0.75	0.4	1	0.825
T2w_Flair_Enhancing_RFE	Bagging	0.7	0.75	0.4	1	0.825

T2w_Flair_Enhancing_RFE	DT	0.7	0.75	0.4	1	0.825
T2w_Flair_Enhancing_RFE	ET	0.7	0.75	0.4	1	0.825
T2w_Flair_Whole_Kbest	QDA	0.7	0.75	0.4	1	0.825
T2w_Flair_Whole_Kbest	SVC	0.7	0.75	0.4	1	0.825
T2w_Flair_Whole_mRmR	AB	0.7	0.75	0.4	1	0.825
T2w_Flair_Whole_mRmR	GB	0.7	0.75	0.4	1	0.825
T2w_Flair_Whole_mRmR	LDA	0.7	0.75	0.4	1	0.825
T2w_Flair_Whole_mRmR	LR	0.7	0.75	0.4	1	0.825
T2w_Flair_Whole_mRmR	MLPC	0.7	0.75	0.4	1	0.825
T2w_Flair_Whole_mRmR	Ridge	0.7	0.75	0.4	1	0.825
T2w_Flair_Whole_mRmR	SVC	0.7	0.75	0.4	1	0.825
T1w_CE_Core_mRmR	AB	0.68571429	0.66666667	0.8	0.57142857	0.72738095
T1w_CE_Core_mRmR	ET	0.68571429	0.66666667	0.8	0.57142857	0.72738095
T1w_CE_Necrosis_RFE	HGB	0.68571429	0.66666667	0.8	0.57142857	0.72738095
T1w_CE_Whole_mRmR	PA	0.68571429	0.66666667	0.8	0.57142857	0.72738095
T2w_Flair_Core_Kbest	ET	0.68571429	0.66666667	0.8	0.57142857	0.72738095
T2w_Flair_Core_RFE	Bagging	0.68571429	0.66666667	0.8	0.57142857	0.72738095
T2w_Flair_Core_RFE	DT	0.68571429	0.66666667	0.8	0.57142857	0.72738095
T2w_Flair_Core_RFE	ET	0.68571429	0.66666667	0.8	0.57142857	0.72738095
T2w_Flair_Core_RFE	GB	0.68571429	0.66666667	0.8	0.57142857	0.72738095
T2w_Flair_Core_RFE	RF	0.68571429	0.66666667	0.8	0.57142857	0.72738095
T2w_Flair_Enhancing_Kbest	Bagging	0.68571429	0.66666667	0.8	0.57142857	0.72738095
T2w_Flair_Enhancing_Kbest	DT	0.68571429	0.66666667	0.8	0.57142857	0.72738095
T2w_Flair_Enhancing_Kbest	ET	0.68571429	0.66666667	0.8	0.57142857	0.72738095
T2w_Flair_Enhancing_Kbest	GB	0.68571429	0.66666667	0.8	0.57142857	0.72738095
T2w_Flair_Enhancing_Kbest	RF	0.68571429	0.66666667	0.8	0.57142857	0.72738095
T2w_Flair_Enhancing_mRmR	AB	0.68571429	0.66666667	0.8	0.57142857	0.72738095
T2w_Flair_Enhancing_mRmR	DT	0.68571429	0.66666667	0.8	0.57142857	0.72738095
T2w_Flair_Enhancing_mRmR	GB	0.68571429	0.66666667	0.8	0.57142857	0.72738095
T2w_Flair_Enhancing_mRmR	HGB	0.68571429	0.66666667	0.8	0.57142857	0.72738095
T2w_Flair_Enhancing_RFE	PA	0.68571429	0.66666667	0.8	0.57142857	0.72738095
T1w_CE_Core_mRmR	KN	0.65714286	0.66666667	0.6	0.71428571	0.68333333
T1w_CE_Edema_mRmR	DT	0.65714286	0.66666667	0.6	0.71428571	0.68333333
T1w_CE_Enhancing_mRmR	KN	0.65714286	0.66666667	0.6	0.71428571	0.68333333
T1w_CE_Necrosis_Kbest	DT	0.65714286	0.66666667	0.6	0.71428571	0.68333333
T1w_CE_Necrosis_Kbest	ET	0.65714286	0.66666667	0.6	0.71428571	0.68333333
T1w_CE_Necrosis_Kbest	KN	0.65714286	0.66666667	0.6	0.71428571	0.68333333
T1w_CE_Necrosis_Kbest	LDA	0.65714286	0.66666667	0.6	0.71428571	0.68333333
T1w_CE_Necrosis_Kbest	LR	0.65714286	0.66666667	0.6	0.71428571	0.68333333
T1w_CE_Necrosis_Kbest	MLPC	0.65714286	0.66666667	0.6	0.71428571	0.68333333

T1w_CE_Necrosis_Kbest	QDA	0.65714286	0.66666667	0.6	0.71428571	0.68333333
T1w_CE_Necrosis_Kbest	RF	0.65714286	0.66666667	0.6	0.71428571	0.68333333
T1w_CE_Necrosis_Kbest	Ridge	0.65714286	0.66666667	0.6	0.71428571	0.68333333
T1w_CE_Necrosis_mRmR	ET	0.65714286	0.66666667	0.6	0.71428571	0.68333333
T1w_CE_Necrosis_mRmR	GNB	0.65714286	0.66666667	0.6	0.71428571	0.68333333
T1w_CE_Necrosis_mRmR	GB	0.65714286	0.66666667	0.6	0.71428571	0.68333333
T1w_CE_Necrosis_mRmR	HGB	0.65714286	0.66666667	0.6	0.71428571	0.68333333
T1w_CE_Necrosis_mRmR	MLPC	0.65714286	0.66666667	0.6	0.71428571	0.68333333
T1w_CE_Necrosis_mRmR	NC	0.65714286	0.66666667	0.6	0.71428571	0.68333333
T1w_CE_Necrosis_mRmR	QDA	0.65714286	0.66666667	0.6	0.71428571	0.68333333
T1w_CE_Necrosis_mRmR	RF	0.65714286	0.66666667	0.6	0.71428571	0.68333333
T1w_CE_Whole_mRmR	GB	0.65714286	0.66666667	0.6	0.71428571	0.68333333
T1w_CE_Whole_mRmR	HGB	0.65714286	0.66666667	0.6	0.71428571	0.68333333
T1w_CE_Whole_RFE	AB	0.65714286	0.66666667	0.6	0.71428571	0.68333333
T2w_Flair_Core_Kbest	GB	0.65714286	0.66666667	0.6	0.71428571	0.68333333
T2w_Flair_Edema_mRmR	PA	0.65714286	0.66666667	0.6	0.71428571	0.68333333
T2w_Flair_Enhancing_Kbest	KN	0.65714286	0.66666667	0.6	0.71428571	0.68333333
T2w_Flair_Enhancing_Kbest	QDA	0.65714286	0.66666667	0.6	0.71428571	0.68333333
T2w_Flair_Enhancing_Kbest	SVC	0.65714286	0.66666667	0.6	0.71428571	0.68333333
T2w_Flair_Enhancing_mRmR	HGB	0.65714286	0.66666667	0.6	0.71428571	0.68333333
T2w_Flair_Enhancing_RFE	Bagging	0.65714286	0.66666667	0.6	0.71428571	0.68333333
T2w_Flair_Whole_Kbest	PA	0.65714286	0.66666667	0.6	0.71428571	0.68333333
T2w_Flair_Whole_mRmR	Bagging	0.65714286	0.66666667	0.6	0.71428571	0.68333333
T2w_Flair_Whole_mRmR	GNB	0.65714286	0.66666667	0.6	0.71428571	0.68333333
T2w_Flair_Whole_mRmR	NC	0.65714286	0.66666667	0.6	0.71428571	0.68333333
T2w_Flair_Whole_RFE	AB	0.65714286	0.66666667	0.6	0.71428571	0.68333333
T2w_Flair_Whole_RFE	DT	0.65714286	0.66666667	0.6	0.71428571	0.68333333
T1w_CE_Necrosis_RFE	QDA	0.64285714	0.58333333	1	0.28571429	0.75
T2w_Flair_Whole_RFE	PA	0.64285714	0.58333333	1	0.28571429	0.75
T1w_CE_Core_mRmR	LDA	0.62857143	0.66666667	0.4	0.85714286	0.65833333
T1w_CE_Edema_mRmR	AB	0.62857143	0.66666667	0.4	0.85714286	0.65833333
T1w_CE_Edema_mRmR	HGB	0.62857143	0.66666667	0.4	0.85714286	0.65833333
T1w_CE_Necrosis_mRmR	AB	0.62857143	0.66666667	0.4	0.85714286	0.65833333
T1w_CE_Necrosis_mRmR	Bagging	0.62857143	0.66666667	0.4	0.85714286	0.65833333
T1w_CE_Whole_mRmR	Bagging	0.62857143	0.66666667	0.4	0.85714286	0.65833333
T1w_CE_Whole_mRmR	ET	0.62857143	0.66666667	0.4	0.85714286	0.65833333
T2w_Flair_Core_Kbest	KN	0.62857143	0.66666667	0.4	0.85714286	0.65833333
T2w_Flair_Core_RFE	LR	0.62857143	0.66666667	0.4	0.85714286	0.65833333
T2w_Flair_Edema_Kbest	AB	0.62857143	0.66666667	0.4	0.85714286	0.65833333
T2w_Flair_Edema_Kbest	DT	0.62857143	0.66666667	0.4	0.85714286	0.65833333

T2w_Flair_Edema_mRmR	AB	0.62857143	0.66666667	0.4	0.85714286	0.65833333
T2w_Flair_Enhancing_Kbest	GNB	0.62857143	0.66666667	0.4	0.85714286	0.65833333
T2w_Flair_Enhancing_Kbest	MLPC	0.62857143	0.66666667	0.4	0.85714286	0.65833333
T2w_Flair_Enhancing_Kbest	GNB	0.62857143	0.66666667	0.4	0.85714286	0.65833333
T2w_Flair_Enhancing_Kbest	QDA	0.62857143	0.66666667	0.4	0.85714286	0.65833333
T2w_Flair_Enhancing_mRmR	KN	0.62857143	0.66666667	0.4	0.85714286	0.65833333
T2w_Flair_Enhancing_RFE	KN	0.62857143	0.66666667	0.4	0.85714286	0.65833333
T2w_Flair_Enhancing_RFE	AB	0.62857143	0.66666667	0.4	0.85714286	0.65833333
T2w_Flair_Enhancing_RFE	GB	0.62857143	0.66666667	0.4	0.85714286	0.65833333
T2w_Flair_Enhancing_RFE	HGB	0.62857143	0.66666667	0.4	0.85714286	0.65833333
T2w_Flair_Whole_Kbest	GNB	0.62857143	0.66666667	0.4	0.85714286	0.65833333
T2w_Flair_Whole_RFE	KN	0.62857143	0.66666667	0.4	0.85714286	0.65833333
T2w_Flair_Whole_RFE	QDA	0.62857143	0.66666667	0.4	0.85714286	0.65833333
T1w_CE_Core_mRmR	NC	0.61428571	0.58333333	0.8	0.42857143	0.69166667
T1w_CE_Core_mRmR	RF	0.61428571	0.58333333	0.8	0.42857143	0.69166667
T1w_CE_Necrosis_Kbest	Bagging	0.61428571	0.58333333	0.8	0.42857143	0.69166667
T1w_CE_Necrosis_Kbest	GNB	0.61428571	0.58333333	0.8	0.42857143	0.69166667
T1w_CE_Necrosis_RFE	RF	0.61428571	0.58333333	0.8	0.42857143	0.69166667
T2w_Flair_Enhancing_Kbest	NC	0.61428571	0.58333333	0.8	0.42857143	0.69166667
T1w_CE_Edema_mRmR	GNB	0.6	0.66666667	0.2	1	0.76666667
T1w_CE_Edema_mRmR	NC	0.6	0.66666667	0.2	1	0.76666667
T1w_CE_Edema_mRmR	RF	0.6	0.66666667	0.2	1	0.76666667
T1w_CE_Edema_RFE	GB	0.6	0.66666667	0.2	1	0.76666667
T1w_CE_Enhancing_Kbest	NC	0.6	0.66666667	0.2	1	0.76666667
T1w_CE_Enhancing_mRmR	Bagging	0.6	0.66666667	0.2	1	0.76666667
T1w_CE_Enhancing_mRmR	QDA	0.6	0.66666667	0.2	1	0.76666667
T1w_CE_Enhancing_RFE	BNB	0.6	0.66666667	0.2	1	0.76666667
T1w_CE_Necrosis_RFE	LR	0.6	0.66666667	0.2	1	0.76666667
T1w_CE_Whole_mRmR	GNB	0.6	0.66666667	0.2	1	0.76666667
T1w_CE_Whole_mRmR	LR	0.6	0.66666667	0.2	1	0.76666667
T2w_Flair_Core_mRmR	GNB	0.6	0.66666667	0.2	1	0.76666667
T2w_Flair_Core_mRmR	LR	0.6	0.66666667	0.2	1	0.76666667
T2w_Flair_Edema_Kbest	BNB	0.6	0.66666667	0.2	1	0.76666667
T2w_Flair_Edema_mRmR	Bagging	0.6	0.66666667	0.2	1	0.76666667
T2w_Flair_Edema_mRmR	DT	0.6	0.66666667	0.2	1	0.76666667
T2w_Flair_Edema_mRmR	GB	0.6	0.66666667	0.2	1	0.76666667
T2w_Flair_Edema_mRmR	KN	0.6	0.66666667	0.2	1	0.76666667
T2w_Flair_Edema_RFE	MLPC	0.6	0.66666667	0.2	1	0.76666667
T2w_Flair_Edema_RFE	RF	0.6	0.66666667	0.2	1	0.76666667
T2w_Flair_Enhancing_RFE	SVC	0.6	0.66666667	0.2	1	0.76666667

T2w_Flair_Whole_Kbest	BNB	0.6	0.66666667	0.2	1	0.76666667
T2w_Flair_Whole_mRmR	BNB	0.6	0.66666667	0.2	1	0.76666667
T1w_CE_Necrosis_Kbest	GB	0.58571429	0.58333333	0.6	0.57142857	0.63333333
T1w_CE_Necrosis_Kbest	HGB	0.58571429	0.58333333	0.6	0.57142857	0.63333333
T1w_CE_Necrosis_RFE	Bagging	0.58571429	0.58333333	0.6	0.57142857	0.63333333
T2w_Flair_Core_Kbest	Bagging	0.58571429	0.58333333	0.6	0.57142857	0.63333333
T2w_Flair_Core_Kbest	DT	0.58571429	0.58333333	0.6	0.57142857	0.63333333
T2w_Flair_Core_Kbest	RF	0.58571429	0.58333333	0.6	0.57142857	0.63333333
T2w_Flair_Enhancing_Kbest	AB	0.58571429	0.58333333	0.6	0.57142857	0.63333333
T2w_Flair_Enhancing_mRmR	DT	0.58571429	0.58333333	0.6	0.57142857	0.63333333
T2w_Flair_Enhancing_mRmR	Bagging	0.58571429	0.58333333	0.6	0.57142857	0.63333333
T2w_Flair_Enhancing_mRmR	ET	0.58571429	0.58333333	0.6	0.57142857	0.63333333
T2w_Flair_Enhancing_mRmR	RF	0.58571429	0.58333333	0.6	0.57142857	0.63333333
T2w_Flair_Enhancing_RFE	DT	0.58571429	0.58333333	0.6	0.57142857	0.63333333
T2w_Flair_Whole_mRmR	HGB	0.58571429	0.58333333	0.6	0.57142857	0.63333333
T1w_CE_Core_mRmR	DT	0.57142857	0.5	1	0.14285714	0.72727273
T1w_CE_Edema_Kbest	GB	0.55714286	0.58333333	0.4	0.71428571	0.575
T2w_Flair_Core_Kbest	HGB	0.55714286	0.58333333	0.4	0.71428571	0.575
T2w_Flair_Core_mRmR	DT	0.55714286	0.58333333	0.4	0.71428571	0.575
T2w_Flair_Core_mRmR	NC	0.55714286	0.58333333	0.4	0.71428571	0.575
T2w_Flair_Core_mRmR	PA	0.55714286	0.58333333	0.4	0.71428571	0.575
T2w_Flair_Whole_Kbest	LDA	0.55714286	0.58333333	0.4	0.71428571	0.575
T2w_Flair_Whole_Kbest	LR	0.55714286	0.58333333	0.4	0.71428571	0.575
T2w_Flair_Whole_Kbest	MLPC	0.55714286	0.58333333	0.4	0.71428571	0.575
T2w_Flair_Whole_Kbest	NC	0.55714286	0.58333333	0.4	0.71428571	0.575
T2w_Flair_Whole_Kbest	Ridge	0.55714286	0.58333333	0.4	0.71428571	0.575
T2w_Flair_Whole_mRmR	QDA	0.55714286	0.58333333	0.4	0.71428571	0.575
T1w_CE_Edema_mRmR	GB	0.52857143	0.58333333	0.2	0.85714286	0.51666667
T1w_CE_Edema_RFE	KN	0.52857143	0.58333333	0.2	0.85714286	0.51666667
T1w_CE_Enhancing_Kbest	AB	0.52857143	0.58333333	0.2	0.85714286	0.51666667
T1w_CE_Necrosis_RFE	Ridge	0.52857143	0.58333333	0.2	0.85714286	0.51666667
T1w_CE_Whole_mRmR	KN	0.52857143	0.58333333	0.2	0.85714286	0.51666667
T2w_Flair_Core_Kbest	AB	0.52857143	0.58333333	0.2	0.85714286	0.51666667
T2w_Flair_Edema_Kbest	GB	0.52857143	0.58333333	0.2	0.85714286	0.51666667
T2w_Flair_Edema_Kbest	QDA	0.52857143	0.58333333	0.2	0.85714286	0.51666667
T2w_Flair_Edema_Kbest	RF	0.52857143	0.58333333	0.2	0.85714286	0.51666667
T2w_Flair_Edema_mRmR	QDA	0.52857143	0.58333333	0.2	0.85714286	0.51666667
T2w_Flair_Edema_RFE	Bagging	0.52857143	0.58333333	0.2	0.85714286	0.51666667
T2w_Flair_Edema_RFE	BNB	0.52857143	0.58333333	0.2	0.85714286	0.51666667
T2w_Flair_Edema_RFE	KN	0.52857143	0.58333333	0.2	0.85714286	0.51666667

T2w_Flair_Enhancing_Kbest	LDA	0.52857143	0.58333333	0.2	0.85714286	0.51666667
T2w_Flair_Enhancing_mRmR	NC	0.52857143	0.58333333	0.2	0.85714286	0.51666667
T2w_Flair_Enhancing_mRmR	QDA	0.52857143	0.58333333	0.2	0.85714286	0.51666667
T2w_Flair_Enhancing_mRmR	NC	0.52857143	0.58333333	0.2	0.85714286	0.51666667
T2w_Flair_Enhancing_RFE	AB	0.52857143	0.58333333	0.2	0.85714286	0.51666667
T2w_Flair_Enhancing_RFE	QDA	0.52857143	0.58333333	0.2	0.85714286	0.51666667
T1w_CE_Edema_Kbest	HGB	0.51428571	0.5	0.6	0.42857143	0.59761905
T1w_CE_Edema_Kbest	KN	0.51428571	0.5	0.6	0.42857143	0.59761905
T1w_CE_Necrosis_RFE	DT	0.51428571	0.5	0.6	0.42857143	0.59761905
T1w_CE_Necrosis_RFE	GB	0.51428571	0.5	0.6	0.42857143	0.59761905
T1w_CE_Core_Kbest	BNB	0.5	0.58333333	0	1	0.70833333
T1w_CE_Core_Kbest	PA	0.5	0.58333333	0	1	0.70833333
T1w_CE_Core_mRmR	BNB	0.5	0.58333333	0	1	0.70833333
T1w_CE_Core_mRmR	PA	0.5	0.58333333	0	1	0.70833333
T1w_CE_Core_RFE	BNB	0.5	0.58333333	0	1	0.70833333
T1w_CE_Core_RFE	LDA	0.5	0.58333333	0	1	0.70833333
T1w_CE_Core_RFE	LR	0.5	0.58333333	0	1	0.70833333
T1w_CE_Core_RFE	MLPC	0.5	0.58333333	0	1	0.70833333
T1w_CE_Core_RFE	NC	0.5	0.58333333	0	1	0.70833333
T1w_CE_Core_RFE	Ridge	0.5	0.58333333	0	1	0.70833333
T1w_CE_Edema_Kbest	BNB	0.5	0.58333333	0	1	0.70833333
T1w_CE_Edema_mRmR	BNB	0.5	0.58333333	0	1	0.70833333
T1w_CE_Edema_mRmR	LDA	0.5	0.58333333	0	1	0.70833333
T1w_CE_Edema_mRmR	LR	0.5	0.58333333	0	1	0.70833333
T1w_CE_Edema_mRmR	PA	0.5	0.41666667	1	0	0.70833333
T1w_CE_Edema_mRmR	Ridge	0.5	0.58333333	0	1	0.70833333
T1w_CE_Edema_RFE	AB	0.5	0.58333333	0	1	0.70833333
T1w_CE_Edema_RFE	BNB	0.5	0.58333333	0	1	0.70833333
T1w_CE_Edema_RFE	GNB	0.5	0.58333333	0	1	0.70833333
T1w_CE_Edema_RFE	LDA	0.5	0.58333333	0	1	0.70833333
T1w_CE_Edema_RFE	LR	0.5	0.58333333	0	1	0.70833333
T1w_CE_Edema_RFE	MLPC	0.5	0.58333333	0	1	0.70833333
T1w_CE_Edema_RFE	PA	0.5	0.58333333	0	1	0.70833333
T1w_CE_Edema_RFE	QDA	0.5	0.58333333	0	1	0.70833333
T1w_CE_Edema_RFE	Ridge	0.5	0.58333333	0	1	0.70833333
T1w_CE_Edema_RFE	SVC	0.5	0.58333333	0	1	0.70833333
T1w_CE_Enhancing_Kbest	BNB	0.5	0.58333333	0	1	0.70833333
T1w_CE_Enhancing_Kbest	LDA	0.5	0.58333333	0	1	0.70833333
T1w_CE_Enhancing_Kbest	LR	0.5	0.58333333	0	1	0.70833333
T1w_CE_Enhancing_Kbest	MLPC	0.5	0.58333333	0	1	0.70833333

T1w_CE_Enhancing_Kbest	Ridge	0.5	0.58333333	0	1	0.70833333
T1w_CE_Enhancing_Kbest	SVC	0.5	0.58333333	0	1	0.70833333
T1w_CE_Enhancing_mRmR	BNB	0.5	0.58333333	0	1	0.70833333
T1w_CE_Enhancing_mRmR	GNB	0.5	0.58333333	0	1	0.70833333
T1w_CE_Enhancing_mRmR	LDA	0.5	0.58333333	0	1	0.70833333
T1w_CE_Enhancing_mRmR	LR	0.5	0.58333333	0	1	0.70833333
T1w_CE_Enhancing_mRmR	MLPC	0.5	0.58333333	0	1	0.70833333
T1w_CE_Enhancing_mRmR	Ridge	0.5	0.58333333	0	1	0.70833333
T1w_CE_Enhancing_RFE	GNB	0.5	0.58333333	0	1	0.70833333
T1w_CE_Enhancing_RFE	LDA	0.5	0.58333333	0	1	0.70833333
T1w_CE_Enhancing_RFE	LR	0.5	0.58333333	0	1	0.70833333
T1w_CE_Enhancing_RFE	NC	0.5	0.58333333	0	1	0.70833333
T1w_CE_Enhancing_RFE	PA	0.5	0.58333333	0	1	0.70833333
T1w_CE_Enhancing_RFE	Ridge	0.5	0.58333333	0	1	0.70833333
T1w_CE_Necrosis_Kbest	BNB	0.5	0.58333333	0	1	0.70833333
T1w_CE_Necrosis_Kbest	PA	0.5	0.41666667	1	0	0.70833333
T1w_CE_Necrosis_mRmR	BNB	0.5	0.58333333	0	1	0.70833333
T1w_CE_Necrosis_mRmR	PA	0.5	0.58333333	0	1	0.70833333
T1w_CE_Necrosis_RFE	BNB	0.5	0.58333333	0	1	0.70833333
T1w_CE_Whole_Kbest	BNB	0.5	0.58333333	0	1	0.70833333
T1w_CE_Whole_mRmR	BNB	0.5	0.58333333	0	1	0.70833333
T1w_CE_Whole_RFE	BNB	0.5	0.58333333	0	1	0.70833333
T1w_CE_Whole_RFE	LDA	0.5	0.58333333	0	1	0.70833333
T1w_CE_Whole_RFE	LR	0.5	0.58333333	0	1	0.70833333
T1w_CE_Whole_RFE	PA	0.5	0.41666667	1	0	0.70833333
T1w_CE_Whole_RFE	Ridge	0.5	0.58333333	0	1	0.70833333
T2w_Flair_Core_Kbest	BNB	0.5	0.58333333	0	1	0.70833333
T2w_Flair_Core_Kbest	GNB	0.5	0.58333333	0	1	0.70833333
T2w_Flair_Core_Kbest	LDA	0.5	0.58333333	0	1	0.70833333
T2w_Flair_Core_Kbest	LR	0.5	0.58333333	0	1	0.70833333
T2w_Flair_Core_Kbest	MLPC	0.5	0.58333333	0	1	0.70833333
T2w_Flair_Core_Kbest	PA	0.5	0.41666667	1	0	0.70833333
T2w_Flair_Core_Kbest	QDA	0.5	0.58333333	0	1	0.70833333
T2w_Flair_Core_Kbest	Ridge	0.5	0.58333333	0	1	0.70833333
T2w_Flair_Core_Kbest	SVC	0.5	0.58333333	0	1	0.70833333
T2w_Flair_Core_mRmR	BNB	0.5	0.58333333	0	1	0.70833333
T2w_Flair_Core_RFE	BNB	0.5	0.58333333	0	1	0.70833333
T2w_Flair_Core_RFE	NC	0.5	0.41666667	1	0	0.70833333
T2w_Flair_Core_RFE	PA	0.5	0.58333333	0	1	0.70833333
T2w_Flair_Edema_Kbest	LDA	0.5	0.58333333	0	1	0.70833333

T2w_Flair_Edema_Kbest	LR	0.5	0.58333333	0	1	0.70833333
T2w_Flair_Edema_Kbest	MLPC	0.5	0.58333333	0	1	0.70833333
T2w_Flair_Edema_Kbest	NC	0.5	0.58333333	0	1	0.70833333
T2w_Flair_Edema_Kbest	Ridge	0.5	0.58333333	0	1	0.70833333
T2w_Flair_Edema_Kbest	SVC	0.5	0.58333333	0	1	0.70833333
T2w_Flair_Edema_mRmR	BNB	0.5	0.58333333	0	1	0.70833333
T2w_Flair_Edema_mRmR	LDA	0.5	0.58333333	0	1	0.70833333
T2w_Flair_Edema_mRmR	LR	0.5	0.58333333	0	1	0.70833333
T2w_Flair_Edema_mRmR	NC	0.5	0.58333333	0	1	0.70833333
T2w_Flair_Edema_mRmR	Ridge	0.5	0.58333333	0	1	0.70833333
T2w_Flair_Edema_mRmR	SVC	0.5	0.58333333	0	1	0.70833333
T2w_Flair_Edema_RFE	LDA	0.5	0.58333333	0	1	0.70833333
T2w_Flair_Edema_RFE	LR	0.5	0.58333333	0	1	0.70833333
T2w_Flair_Edema_RFE	NC	0.5	0.58333333	0	1	0.70833333
T2w_Flair_Edema_RFE	PA	0.5	0.58333333	0	1	0.70833333
T2w_Flair_Edema_RFE	Ridge	0.5	0.58333333	0	1	0.70833333
T2w_Flair_Edema_RFE	SVC	0.5	0.58333333	0	1	0.70833333
T2w_Flair_Enhancing_Kbest	BNB	0.5	0.58333333	0	1	0.70833333
T2w_Flair_Enhancing_Kbest	LR	0.5	0.58333333	0	1	0.70833333
T2w_Flair_Enhancing_Kbest	PA	0.5	0.58333333	0	1	0.70833333
T2w_Flair_Enhancing_Kbest	BNB	0.5	0.58333333	0	1	0.70833333
T2w_Flair_Enhancing_Kbest	PA	0.5	0.58333333	0	1	0.70833333
T2w_Flair_Enhancing_mRmR	BNB	0.5	0.58333333	0	1	0.70833333
T2w_Flair_Enhancing_mRmR	GNB	0.5	0.58333333	0	1	0.70833333
T2w_Flair_Enhancing_mRmR	LDA	0.5	0.58333333	0	1	0.70833333
T2w_Flair_Enhancing_mRmR	LR	0.5	0.58333333	0	1	0.70833333
T2w_Flair_Enhancing_mRmR	PA	0.5	0.58333333	0	1	0.70833333
T2w_Flair_Enhancing_mRmR	Ridge	0.5	0.58333333	0	1	0.70833333
T2w_Flair_Enhancing_mRmR	BNB	0.5	0.58333333	0	1	0.70833333
T2w_Flair_Enhancing_mRmR	PA	0.5	0.41666667	1	0	0.70833333
T2w_Flair_Enhancing_RFE	BNB	0.5	0.58333333	0	1	0.70833333
T2w_Flair_Enhancing_RFE	LDA	0.5	0.58333333	0	1	0.70833333
T2w_Flair_Enhancing_RFE	LR	0.5	0.58333333	0	1	0.70833333
T2w_Flair_Enhancing_RFE	MLPC	0.5	0.58333333	0	1	0.70833333
T2w_Flair_Enhancing_RFE	PA	0.5	0.41666667	1	0	0.70833333
T2w_Flair_Enhancing_RFE	Ridge	0.5	0.58333333	0	1	0.70833333
T2w_Flair_Enhancing_RFE	BNB	0.5	0.58333333	0	1	0.70833333
T2w_Flair_Enhancing_RFE	GNB	0.5	0.58333333	0	1	0.70833333
T2w_Flair_Enhancing_RFE	LDA	0.5	0.58333333	0	1	0.70833333
T2w_Flair_Enhancing_RFE	LR	0.5	0.58333333	0	1	0.70833333

T2w_Flair_Enhancing_RFE	MLPC	0.5	0.58333333	0	1	0.70833333
T2w_Flair_Enhancing_RFE	Ridge	0.5	0.58333333	0	1	0.70833333
T2w_Flair_Whole_mRmR	PA	0.5	0.58333333	0	1	0.70833333
T2w_Flair_Whole_RFE	BNB	0.5	0.41666667	1	0	0.70833333
T1w_CE_Necrosis_RFE	KN	0.48571429	0.5	0.4	0.57142857	0.525
T2w_Flair_Edema_Kbest	PA	0.47142857	0.41666667	0.8	0.14285714	0.64166667
T1w_CE_Edema_RFE	Bagging	0.45714286	0.5	0.2	0.71428571	0.43333333
T1w_CE_Edema_RFE	DT	0.45714286	0.5	0.2	0.71428571	0.43333333
T1w_CE_Edema_RFE	ET	0.45714286	0.5	0.2	0.71428571	0.43333333
T1w_CE_Edema_RFE	RF	0.45714286	0.5	0.2	0.71428571	0.43333333
T1w_CE_Necrosis_RFE	ET	0.45714286	0.5	0.2	0.71428571	0.43333333
T1w_CE_Necrosis_RFE	LDA	0.45714286	0.5	0.2	0.71428571	0.43333333
T1w_CE_Necrosis_RFE	PA	0.45714286	0.5	0.2	0.71428571	0.43333333
T1w_CE_Edema_RFE	HGB	0.42857143	0.5	0	0.85714286	0.20833333
T1w_CE_Whole_RFE	NC	0.42857143	0.5	0	0.85714286	0.20833333
T2w_Flair_Edema_Kbest	ET	0.42857143	0.5	0	0.85714286	0.20833333
T2w_Flair_Edema_Kbest	KN	0.42857143	0.5	0	0.85714286	0.20833333
T2w_Flair_Edema_RFE	GNB	0.42857143	0.5	0	0.85714286	0.20833333
T2w_Flair_Edema_RFE	QDA	0.42857143	0.5	0	0.85714286	0.20833333
T2w_Flair_Enhancing_Kbest	Ridge	0.42857143	0.5	0	0.85714286	0.20833333
T2w_Flair_Enhancing_Kbest	LDA	0.42857143	0.5	0	0.85714286	0.20833333
T2w_Flair_Enhancing_Kbest	LR	0.42857143	0.5	0	0.85714286	0.20833333
T2w_Flair_Enhancing_Kbest	Ridge	0.42857143	0.5	0	0.85714286	0.20833333
T2w_Flair_Enhancing_mRmR	GNB	0.42857143	0.5	0	0.85714286	0.20833333
T2w_Flair_Enhancing_mRmR	LDA	0.42857143	0.5	0	0.85714286	0.20833333
T2w_Flair_Enhancing_mRmR	LR	0.42857143	0.5	0	0.85714286	0.20833333
T2w_Flair_Enhancing_mRmR	MLPC	0.42857143	0.5	0	0.85714286	0.20833333
T2w_Flair_Enhancing_mRmR	QDA	0.42857143	0.5	0	0.85714286	0.20833333
T2w_Flair_Enhancing_mRmR	Ridge	0.42857143	0.5	0	0.85714286	0.20833333
T2w_Flair_Enhancing_RFE	GNB	0.42857143	0.5	0	0.85714286	0.20833333
T2w_Flair_Enhancing_RFE	NC	0.42857143	0.5	0	0.85714286	0.20833333
T2w_Flair_Enhancing_RFE	QDA	0.42857143	0.5	0	0.85714286	0.20833333
T2w_Flair_Whole_RFE	LDA	0.42857143	0.5	0	0.85714286	0.20833333
T2w_Flair_Whole_RFE	LR	0.42857143	0.5	0	0.85714286	0.20833333
T2w_Flair_Whole_RFE	NC	0.42857143	0.5	0	0.85714286	0.20833333
T2w_Flair_Whole_RFE	Ridge	0.42857143	0.5	0	0.85714286	0.20833333
T1w_CE_Necrosis_mRmR	DT	0.41428571	0.41666667	0.4	0.42857143	0.49166667
T1w_CE_Necrosis_RFE	AB	0.41428571	0.41666667	0.4	0.42857143	0.49166667
T1w_CE_Whole_mRmR	AB	0.41428571	0.41666667	0.4	0.42857143	0.49166667
T1w_CE_Edema_Kbest	Bagging	0.31428571	0.33333333	0.2	0.42857143	0.36666667

T1w_CE_Necrosis_RFE	GNB	0.3	0.25	0.6	0	0.53333333
T1w_CE_Necrosis_RFE	NC	0.27142857	0.25	0.4	0.14285714	0.45
T1w_CE_Edema_Kbest	DT	0.17142857	0.16666667	0.2	0.14285714	0.33809524
T1w_CE_Edema_Kbest	ET	0.17142857	0.16666667	0.2	0.14285714	0.33809524
T1w_CE_Edema_Kbest	RF	0.17142857	0.16666667	0.2	0.14285714	0.33809524

Table S3. The complete result of multi-machine learning algorithms, feature selection, and multi-segmentation approaches in discriminating IDH-mutant grade-4 astrocytomas from IDH wild-type GBMs of original data.

**Approach**	**ML**	**AUC**	**ACC**	**SEN**	**SPE**	**PR_AUC**
Or_T1w_CE_Core_Kbest	AB	0.92857143	0.91666667	1	0.85714286	0.91666667
Or_T1w_CE_Core_Kbest	KN	0.92857143	0.91666667	1	0.85714286	0.91666667
Or_T1w_CE_Core_RFE	LR	0.92857143	0.91666667	1	0.85714286	0.91666667
Or_T1w_CE_Core_RFE	MLPC	0.92857143	0.91666667	1	0.85714286	0.91666667
Or_T1w_CE_Core_RFE	SVC	0.92857143	0.91666667	1	0.85714286	0.91666667
Or_T1w_CE_Core_RFE	HGB	0.92857143	0.91666667	1	0.85714286	0.91666667
Or_T1w_CE_Core_RFE	HGB	0.92857143	0.91666667	1	0.85714286	0.91666667
Or_T1w_CE_Core_RFE	LDA	0.92857143	0.91666667	1	0.85714286	0.91666667
Or_T1w_CE_Core_RFE	MLPC	0.92857143	0.91666667	1	0.85714286	0.91666667
Or_T1w_CE_Core_RFE	DT	0.92857143	0.91666667	1	0.85714286	0.91666667
Or_T1w_CE_Core_RFE	DT	0.92857143	0.91666667	1	0.85714286	0.91666667
Or_T1w_CE_Core_RFE	GB	0.92857143	0.91666667	1	0.85714286	0.91666667
Or_T1w_CE_Core_RFE	RF	0.92857143	0.91666667	1	0.85714286	0.91666667
Or_T1w_CE_Core_RFE	RF	0.92857143	0.91666667	1	0.85714286	0.91666667
Or_T1w_CE_Core_RFE	Bagging	0.92857143	0.91666667	1	0.85714286	0.91666667
Or_T1w_CE_Core_RFE	ET	0.92857143	0.91666667	1	0.85714286	0.91666667
Or_T1w_CE_Core_RFE	GNB	0.92857143	0.91666667	1	0.85714286	0.91666667
Or_T1w_CE_Core_RFE	GB	0.92857143	0.91666667	1	0.85714286	0.91666667
Or_T1w_CE_Core_RFE	KN	0.92857143	0.91666667	1	0.85714286	0.91666667
Or_T1w_CE_Core_RFE	MLPC	0.92857143	0.91666667	1	0.85714286	0.91666667
Or_T1w_CE_Core_mRmR	NC	0.92857143	0.91666667	1	0.85714286	0.91666667
Or_T1w_CE_Core_mRmR	PA	0.92857143	0.91666667	1	0.85714286	0.91666667
Or_T1w_CE_Core_mRmR	QDA	0.92857143	0.91666667	1	0.85714286	0.91666667
Or_T1w_CE_Core_mRmR	RF	0.92857143	0.91666667	1	0.85714286	0.91666667
Or_T1w_CE_Core_mRmR	Bagging	0.9	0.91666667	0.8	1	0.94166667
Or_T1w_CE_Core_mRmR	DT	0.9	0.91666667	0.8	1	0.94166667
Or_T1w_CE_Core_mRmR	GB	0.9	0.91666667	0.8	1	0.94166667
Or_T1w_CE_Core_mRmR	HGB	0.9	0.91666667	0.8	1	0.94166667
Or_T1w_CE_Core_mRmR	PA	0.9	0.91666667	0.8	1	0.94166667

Or_T1w_CE_Core_mRmR	Ridge	0.9	0.91666667	0.8	1	0.94166667
Or_T1w_CE_Core_mRmR	AB	0.9	0.91666667	0.8	1	0.94166667
Or_T1w_CE_Core_mRmR	Bagging	0.9	0.91666667	0.8	1	0.94166667
Or_T1w_CE_Core_mRmR	DT	0.9	0.91666667	0.8	1	0.94166667
Or_T1w_CE_Core_mRmR	ET	0.9	0.91666667	0.8	1	0.94166667
Or_T1w_CE_Core_mRmR	GB	0.9	0.91666667	0.8	1	0.94166667
Or_T1w_CE_Core_mRmR	HGB	0.9	0.91666667	0.8	1	0.94166667
Or_T1w_CE_Core_mRmR	LDA	0.9	0.91666667	0.8	1	0.94166667
Or_T1w_CE_Core_mRmR	PA	0.9	0.91666667	0.8	1	0.94166667
Or_T1w_CE_Edema_Kbest	RF	0.9	0.91666667	0.8	1	0.94166667
Or_T1w_CE_Edema_Kbest	Ridge	0.9	0.91666667	0.8	1	0.94166667
Or_T1w_CE_Edema_Kbest	AB	0.9	0.91666667	0.8	1	0.94166667
Or_T1w_CE_Edema_Kbest	Bagging	0.9	0.91666667	0.8	1	0.94166667
Or_T1w_CE_Edema_Kbest	DT	0.9	0.91666667	0.8	1	0.94166667
Or_T1w_CE_Edema_Kbest	GB	0.9	0.91666667	0.8	1	0.94166667
Or_T1w_CE_Edema_Kbest	HGB	0.9	0.91666667	0.8	1	0.94166667
Or_T1w_CE_Edema_Kbest	PA	0.9	0.91666667	0.8	1	0.94166667
Or_T1w_CE_Edema_Kbest	Ridge	0.9	0.91666667	0.8	1	0.94166667
Or_T1w_CE_Edema_Kbest	AB	0.9	0.91666667	0.8	1	0.94166667
Or_T1w_CE_Edema_Kbest	Bagging	0.9	0.91666667	0.8	1	0.94166667
Or_T1w_CE_Edema_Kbest	DT	0.9	0.91666667	0.8	1	0.94166667
Or_T1w_CE_Edema_Kbest	ET	0.9	0.91666667	0.8	1	0.94166667
Or_T1w_CE_Edema_Kbest	GNB	0.9	0.91666667	0.8	1	0.94166667
Or_T1w_CE_Edema_Kbest	GB	0.9	0.91666667	0.8	1	0.94166667
Or_T1w_CE_Edema_Kbest	KN	0.9	0.91666667	0.8	1	0.94166667
Or_T1w_CE_Edema_Kbest	LDA	0.9	0.91666667	0.8	1	0.94166667
Or_T1w_CE_Edema_Kbest	MLPC	0.9	0.91666667	0.8	1	0.94166667
Or_T1w_CE_Edema_RFE	NC	0.9	0.91666667	0.8	1	0.94166667
Or_T1w_CE_Edema_RFE	PA	0.9	0.91666667	0.8	1	0.94166667
Or_T1w_CE_Edema_RFE	QDA	0.9	0.91666667	0.8	1	0.94166667
Or_T1w_CE_Edema_RFE	RF	0.9	0.91666667	0.8	1	0.94166667
Or_T1w_CE_Edema_RFE	Ridge	0.9	0.91666667	0.8	1	0.94166667
Or_T1w_CE_Edema_RFE	SVC	0.9	0.91666667	0.8	1	0.94166667
Or_T1w_CE_Edema_RFE	AB	0.9	0.91666667	0.8	1	0.94166667
Or_T1w_CE_Edema_RFE	Bagging	0.9	0.91666667	0.8	1	0.94166667
Or_T1w_CE_Edema_RFE	DT	0.9	0.91666667	0.8	1	0.94166667
Or_T1w_CE_Edema_RFE	ET	0.9	0.91666667	0.8	1	0.94166667
Or_T1w_CE_Edema_RFE	GNB	0.9	0.91666667	0.8	1	0.94166667
Or_T1w_CE_Edema_RFE	GB	0.9	0.91666667	0.8	1	0.94166667
Or_T1w_CE_Edema_RFE	KN	0.9	0.91666667	0.8	1	0.94166667
Or_T1w_CE_Edema_RFE	LDA	0.9	0.91666667	0.8	1	0.94166667
Or_T1w_CE_Edema_RFE	MLPC	0.9	0.91666667	0.8	1	0.94166667
Or_T1w_CE_Edema_RFE	NC	0.9	0.91666667	0.8	1	0.94166667
Or_T1w_CE_Edema_RFE	PA	0.9	0.91666667	0.8	1	0.94166667

Or_T1w_CE_Edema_RFE	QDA	0.9	0.91666667	0.8	1	0.94166667
Or_T1w_CE_Edema_mRmR	RF	0.9	0.91666667	0.8	1	0.94166667
Or_T1w_CE_Edema_mRmR	Ridge	0.9	0.91666667	0.8	1	0.94166667
Or_T1w_CE_Edema_mRmR	SVC	0.9	0.91666667	0.8	1	0.94166667
Or_T1w_CE_Edema_mRmR	AB	0.9	0.91666667	0.8	1	0.94166667
Or_T1w_CE_Edema_mRmR	DT	0.9	0.91666667	0.8	1	0.94166667
Or_T1w_CE_Edema_mRmR	ET	0.9	0.91666667	0.8	1	0.94166667
Or_T1w_CE_Edema_mRmR	GNB	0.9	0.91666667	0.8	1	0.94166667
Or_T1w_CE_Edema_mRmR	GB	0.9	0.91666667	0.8	1	0.94166667
Or_T1w_CE_Edema_mRmR	MLPC	0.9	0.91666667	0.8	1	0.94166667
Or_T1w_CE_Edema_mRmR	NC	0.9	0.91666667	0.8	1	0.94166667
Or_T1w_CE_Edema_mRmR	PA	0.9	0.91666667	0.8	1	0.94166667
Or_T1w_CE_Edema_mRmR	QDA	0.9	0.91666667	0.8	1	0.94166667
Or_T1w_CE_Edema_mRmR	RF	0.9	0.91666667	0.8	1	0.94166667
Or_T1w_CE_Edema_mRmR	SVC	0.9	0.91666667	0.8	1	0.94166667
Or_T1w_CE_Edema_mRmR	DT	0.9	0.91666667	0.8	1	0.94166667
Or_T1w_CE_Edema_mRmR	ET	0.9	0.91666667	0.8	1	0.94166667
Or_T1w_CE_Edema_mRmR	LDA	0.9	0.91666667	0.8	1	0.94166667
Or_T1w_CE_Edema_mRmR	PA	0.9	0.91666667	0.8	1	0.94166667
Or_T1w_CE_Enhancing_Kbest	QDA	0.9	0.91666667	0.8	1	0.94166667
Or_T1w_CE_Enhancing_Kbest	RF	0.9	0.91666667	0.8	1	0.94166667
Or_T1w_CE_Enhancing_Kbest	Ridge	0.9	0.91666667	0.8	1	0.94166667
Or_T1w_CE_Enhancing_Kbest	AB	0.9	0.91666667	0.8	1	0.94166667
Or_T1w_CE_Enhancing_Kbest	Bagging	0.9	0.91666667	0.8	1	0.94166667
Or_T1w_CE_Enhancing_Kbest	DT	0.9	0.91666667	0.8	1	0.94166667
Or_T1w_CE_Enhancing_Kbest	ET	0.9	0.91666667	0.8	1	0.94166667
Or_T1w_CE_Enhancing_Kbest	GNB	0.9	0.91666667	0.8	1	0.94166667
Or_T1w_CE_Enhancing_Kbest	GB	0.9	0.91666667	0.8	1	0.94166667
Or_T1w_CE_Enhancing_Kbest	KN	0.9	0.91666667	0.8	1	0.94166667
Or_T1w_CE_Enhancing_Kbest	LDA	0.9	0.91666667	0.8	1	0.94166667
Or_T1w_CE_Enhancing_Kbest	LR	0.9	0.91666667	0.8	1	0.94166667
Or_T1w_CE_Enhancing_Kbest	MLPC	0.9	0.91666667	0.8	1	0.94166667
Or_T1w_CE_Enhancing_Kbest	PA	0.9	0.91666667	0.8	1	0.94166667
Or_T1w_CE_Enhancing_Kbest	QDA	0.9	0.91666667	0.8	1	0.94166667
Or_T1w_CE_Enhancing_Kbest	RF	0.9	0.91666667	0.8	1	0.94166667
Or_T1w_CE_Enhancing_Kbest	Ridge	0.9	0.91666667	0.8	1	0.94166667
Or_T1w_CE_Enhancing_Kbest	SVC	0.9	0.91666667	0.8	1	0.94166667
Or_T1w_CE_Enhancing_RFE	AB	0.9	0.91666667	0.8	1	0.94166667
Or_T1w_CE_Enhancing_RFE	Bagging	0.9	0.91666667	0.8	1	0.94166667
Or_T1w_CE_Enhancing_RFE	DT	0.9	0.91666667	0.8	1	0.94166667
Or_T1w_CE_Enhancing_RFE	ET	0.9	0.91666667	0.8	1	0.94166667
Or_T1w_CE_Enhancing_RFE	GNB	0.9	0.91666667	0.8	1	0.94166667
Or_T1w_CE_Enhancing_RFE	GB	0.9	0.91666667	0.8	1	0.94166667
Or_T1w_CE_Enhancing_RFE	KN	0.9	0.91666667	0.8	1	0.94166667

Or_T1w_CE_Enhancing_RFE	MLPC	0.9	0.91666667	0.8	1	0.94166667
Or_T1w_CE_Enhancing_RFE	QDA	0.9	0.91666667	0.8	1	0.94166667
Or_T1w_CE_Enhancing_RFE	RF	0.9	0.91666667	0.8	1	0.94166667
Or_T1w_CE_Enhancing_RFE	Ridge	0.9	0.91666667	0.8	1	0.94166667
Or_T1w_CE_Enhancing_RFE	SVC	0.9	0.91666667	0.8	1	0.94166667
Or_T1w_CE_Enhancing_RFE	AB	0.9	0.91666667	0.8	1	0.94166667
Or_T1w_CE_Enhancing_RFE	DT	0.9	0.91666667	0.8	1	0.94166667
Or_T1w_CE_Enhancing_RFE	GB	0.9	0.91666667	0.8	1	0.94166667
Or_T1w_CE_Enhancing_RFE	HGB	0.9	0.91666667	0.8	1	0.94166667
Or_T1w_CE_Enhancing_RFE	AB	0.9	0.91666667	0.8	1	0.94166667
Or_T1w_CE_Enhancing_RFE	DT	0.9	0.91666667	0.8	1	0.94166667
Or_T1w_CE_Enhancing_mRmR	GNB	0.9	0.91666667	0.8	1	0.94166667
Or_T1w_CE_Enhancing_mRmR	LDA	0.9	0.91666667	0.8	1	0.94166667
Or_T1w_CE_Enhancing_mRmR	MLPC	0.9	0.91666667	0.8	1	0.94166667
Or_T1w_CE_Enhancing_mRmR	PA	0.9	0.91666667	0.8	1	0.94166667
Or_T1w_CE_Enhancing_mRmR	QDA	0.9	0.91666667	0.8	1	0.94166667
Or_T1w_CE_Enhancing_mRmR	SVC	0.9	0.91666667	0.8	1	0.94166667
Or_T1w_CE_Enhancing_mRmR	HGB	0.9	0.91666667	0.8	1	0.94166667
Or_T1w_CE_Enhancing_mRmR	PA	0.9	0.91666667	0.8	1	0.94166667
Or_T1w_CE_Enhancing_mRmR	Bagging	0.9	0.91666667	0.8	1	0.94166667
Or_T1w_CE_Enhancing_mRmR	DT	0.9	0.91666667	0.8	1	0.94166667
Or_T1w_CE_Enhancing_mRmR	GB	0.9	0.91666667	0.8	1	0.94166667
Or_T1w_CE_Enhancing_mRmR	Bagging	0.9	0.91666667	0.8	1	0.94166667
Or_T1w_CE_Enhancing_mRmR	HGB	0.9	0.91666667	0.8	1	0.94166667
Or_T1w_CE_Enhancing_mRmR	RF	0.9	0.91666667	0.8	1	0.94166667
Or_T1w_CE_Enhancing_mRmR	DT	0.9	0.91666667	0.8	1	0.94166667
Or_T1w_CE_Enhancing_mRmR	ET	0.9	0.91666667	0.8	1	0.94166667
Or_T1w_CE_Enhancing_mRmR	LDA	0.9	0.91666667	0.8	1	0.94166667
Or_T1w_CE_Enhancing_mRmR	RF	0.9	0.91666667	0.8	1	0.94166667
Or_T1w_CE_Necrosis_Kbest	NC	0.85714286	0.83333333	1	0.71428571	0.85714286
Or_T1w_CE_Necrosis_Kbest	QDA	0.85714286	0.83333333	1	0.71428571	0.85714286
Or_T1w_CE_Necrosis_Kbest	PA	0.85714286	0.83333333	1	0.71428571	0.85714286
Or_T1w_CE_Necrosis_Kbest	GB	0.85714286	0.83333333	1	0.71428571	0.85714286
Or_T1w_CE_Necrosis_Kbest	KN	0.82857143	0.83333333	0.8	0.85714286	0.84166667
Or_T1w_CE_Necrosis_Kbest	Bagging	0.82857143	0.83333333	0.8	0.85714286	0.84166667
Or_T1w_CE_Necrosis_Kbest	LDA	0.82857143	0.83333333	0.8	0.85714286	0.84166667
Or_T1w_CE_Necrosis_Kbest	NC	0.82857143	0.83333333	0.8	0.85714286	0.84166667
Or_T1w_CE_Necrosis_Kbest	GNB	0.82857143	0.83333333	0.8	0.85714286	0.84166667
Or_T1w_CE_Necrosis_Kbest	KN	0.82857143	0.83333333	0.8	0.85714286	0.84166667
Or_T1w_CE_Necrosis_Kbest	NC	0.82857143	0.83333333	0.8	0.85714286	0.84166667
Or_T1w_CE_Necrosis_Kbest	PA	0.82857143	0.83333333	0.8	0.85714286	0.84166667
Or_T1w_CE_Necrosis_Kbest	QDA	0.82857143	0.83333333	0.8	0.85714286	0.84166667
Or_T1w_CE_Necrosis_Kbest	SVC	0.82857143	0.83333333	0.8	0.85714286	0.84166667
Or_T1w_CE_Necrosis_Kbest	AB	0.82857143	0.83333333	0.8	0.85714286	0.84166667

Or_T1w_CE_Necrosis_Kbest	Bagging	0.82857143	0.83333333	0.8	0.85714286	0.84166667
Or_T1w_CE_Necrosis_Kbest	DT	0.82857143	0.83333333	0.8	0.85714286	0.84166667
Or_T1w_CE_Necrosis_Kbest	ET	0.82857143	0.83333333	0.8	0.85714286	0.84166667
Or_T1w_CE_Necrosis_RFE	GNB	0.82857143	0.83333333	0.8	0.85714286	0.84166667
Or_T1w_CE_Necrosis_RFE	GB	0.82857143	0.83333333	0.8	0.85714286	0.84166667
Or_T1w_CE_Necrosis_RFE	KN	0.82857143	0.83333333	0.8	0.85714286	0.84166667
Or_T1w_CE_Necrosis_RFE	LDA	0.82857143	0.83333333	0.8	0.85714286	0.84166667
Or_T1w_CE_Necrosis_RFE	MLPC	0.82857143	0.83333333	0.8	0.85714286	0.84166667
Or_T1w_CE_Necrosis_RFE	NC	0.82857143	0.83333333	0.8	0.85714286	0.84166667
Or_T1w_CE_Necrosis_RFE	QDA	0.82857143	0.83333333	0.8	0.85714286	0.84166667
Or_T1w_CE_Necrosis_RFE	RF	0.82857143	0.83333333	0.8	0.85714286	0.84166667
Or_T1w_CE_Necrosis_RFE	Ridge	0.82857143	0.83333333	0.8	0.85714286	0.84166667
Or_T1w_CE_Necrosis_RFE	SVC	0.82857143	0.83333333	0.8	0.85714286	0.84166667
Or_T1w_CE_Necrosis_RFE	AB	0.82857143	0.83333333	0.8	0.85714286	0.84166667
Or_T1w_CE_Necrosis_RFE	Bagging	0.82857143	0.83333333	0.8	0.85714286	0.84166667
Or_T1w_CE_Necrosis_RFE	DT	0.82857143	0.83333333	0.8	0.85714286	0.84166667
Or_T1w_CE_Necrosis_RFE	ET	0.82857143	0.83333333	0.8	0.85714286	0.84166667
Or_T1w_CE_Necrosis_RFE	GNB	0.82857143	0.83333333	0.8	0.85714286	0.84166667
Or_T1w_CE_Necrosis_RFE	GB	0.82857143	0.83333333	0.8	0.85714286	0.84166667
Or_T1w_CE_Necrosis_RFE	KN	0.82857143	0.83333333	0.8	0.85714286	0.84166667
Or_T1w_CE_Necrosis_RFE	LDA	0.82857143	0.83333333	0.8	0.85714286	0.84166667
Or_T1w_CE_Necrosis_mRmR	MLPC	0.82857143	0.83333333	0.8	0.85714286	0.84166667
Or_T1w_CE_Necrosis_mRmR	NC	0.82857143	0.83333333	0.8	0.85714286	0.84166667
Or_T1w_CE_Necrosis_mRmR	PA	0.82857143	0.83333333	0.8	0.85714286	0.84166667
Or_T1w_CE_Necrosis_mRmR	QDA	0.82857143	0.83333333	0.8	0.85714286	0.84166667
Or_T1w_CE_Necrosis_mRmR	RF	0.82857143	0.83333333	0.8	0.85714286	0.84166667
Or_T1w_CE_Necrosis_mRmR	Ridge	0.82857143	0.83333333	0.8	0.85714286	0.84166667
Or_T1w_CE_Necrosis_mRmR	SVC	0.82857143	0.83333333	0.8	0.85714286	0.84166667
Or_T1w_CE_Necrosis_mRmR	AB	0.82857143	0.83333333	0.8	0.85714286	0.84166667
Or_T1w_CE_Necrosis_mRmR	Bagging	0.82857143	0.83333333	0.8	0.85714286	0.84166667
Or_T1w_CE_Necrosis_mRmR	ET	0.82857143	0.83333333	0.8	0.85714286	0.84166667
Or_T1w_CE_Necrosis_mRmR	GNB	0.82857143	0.83333333	0.8	0.85714286	0.84166667
Or_T1w_CE_Necrosis_mRmR	GB	0.82857143	0.83333333	0.8	0.85714286	0.84166667
Or_T1w_CE_Necrosis_mRmR	KN	0.82857143	0.83333333	0.8	0.85714286	0.84166667
Or_T1w_CE_Necrosis_mRmR	LDA	0.82857143	0.83333333	0.8	0.85714286	0.84166667
Or_T1w_CE_Necrosis_mRmR	LR	0.82857143	0.83333333	0.8	0.85714286	0.84166667
Or_T1w_CE_Necrosis_mRmR	MLPC	0.82857143	0.83333333	0.8	0.85714286	0.84166667
Or_T1w_CE_Necrosis_mRmR	NC	0.82857143	0.83333333	0.8	0.85714286	0.84166667
Or_T1w_CE_Necrosis_mRmR	QDA	0.82857143	0.83333333	0.8	0.85714286	0.84166667
Or_T1w_CE_Whole_Kbest	RF	0.82857143	0.83333333	0.8	0.85714286	0.84166667
Or_T1w_CE_Whole_Kbest	Ridge	0.82857143	0.83333333	0.8	0.85714286	0.84166667
Or_T1w_CE_Whole_Kbest	SVC	0.82857143	0.83333333	0.8	0.85714286	0.84166667
Or_T1w_CE_Whole_Kbest	LDA	0.82857143	0.83333333	0.8	0.85714286	0.84166667
Or_T1w_CE_Whole_Kbest	Bagging	0.82857143	0.83333333	0.8	0.85714286	0.84166667

Or_T1w_CE_Whole_Kbest	ET	0.82857143	0.83333333	0.8	0.85714286	0.84166667
Or_T1w_CE_Whole_Kbest	NC	0.82857143	0.83333333	0.8	0.85714286	0.84166667
Or_T1w_CE_Whole_Kbest	SVC	0.82857143	0.83333333	0.8	0.85714286	0.84166667
Or_T1w_CE_Whole_Kbest	Bagging	0.82857143	0.83333333	0.8	0.85714286	0.84166667
Or_T1w_CE_Whole_Kbest	ET	0.82857143	0.83333333	0.8	0.85714286	0.84166667
Or_T1w_CE_Whole_Kbest	GNB	0.82857143	0.83333333	0.8	0.85714286	0.84166667
Or_T1w_CE_Whole_Kbest	NC	0.82857143	0.83333333	0.8	0.85714286	0.84166667
Or_T1w_CE_Whole_Kbest	AB	0.82857143	0.83333333	0.8	0.85714286	0.84166667
Or_T1w_CE_Whole_Kbest	Bagging	0.82857143	0.83333333	0.8	0.85714286	0.84166667
Or_T1w_CE_Whole_Kbest	DT	0.82857143	0.83333333	0.8	0.85714286	0.84166667
Or_T1w_CE_Whole_Kbest	ET	0.82857143	0.83333333	0.8	0.85714286	0.84166667
Or_T1w_CE_Whole_Kbest	GNB	0.82857143	0.83333333	0.8	0.85714286	0.84166667
Or_T1w_CE_Whole_Kbest	GB	0.82857143	0.83333333	0.8	0.85714286	0.84166667
Or_T1w_CE_Whole_RFE	KN	0.82857143	0.83333333	0.8	0.85714286	0.84166667
Or_T1w_CE_Whole_RFE	LDA	0.82857143	0.83333333	0.8	0.85714286	0.84166667
Or_T1w_CE_Whole_RFE	MLPC	0.82857143	0.83333333	0.8	0.85714286	0.84166667
Or_T1w_CE_Whole_RFE	NC	0.82857143	0.83333333	0.8	0.85714286	0.84166667
Or_T1w_CE_Whole_RFE	PA	0.82857143	0.83333333	0.8	0.85714286	0.84166667
Or_T1w_CE_Whole_RFE	QDA	0.82857143	0.83333333	0.8	0.85714286	0.84166667
Or_T1w_CE_Whole_RFE	Ridge	0.82857143	0.83333333	0.8	0.85714286	0.84166667
Or_T1w_CE_Whole_RFE	SVC	0.82857143	0.83333333	0.8	0.85714286	0.84166667
Or_T1w_CE_Whole_RFE	AB	0.82857143	0.83333333	0.8	0.85714286	0.84166667
Or_T1w_CE_Whole_RFE	DT	0.82857143	0.83333333	0.8	0.85714286	0.84166667
Or_T1w_CE_Whole_RFE	HGB	0.82857143	0.83333333	0.8	0.85714286	0.84166667
Or_T1w_CE_Whole_RFE	LDA	0.82857143	0.83333333	0.8	0.85714286	0.84166667
Or_T1w_CE_Whole_RFE	LR	0.82857143	0.83333333	0.8	0.85714286	0.84166667
Or_T1w_CE_Whole_RFE	Ridge	0.82857143	0.83333333	0.8	0.85714286	0.84166667
Or_T1w_CE_Whole_RFE	SVC	0.82857143	0.83333333	0.8	0.85714286	0.84166667
Or_T1w_CE_Whole_RFE	AB	0.82857143	0.83333333	0.8	0.85714286	0.84166667
Or_T1w_CE_Whole_RFE	Bagging	0.82857143	0.83333333	0.8	0.85714286	0.84166667
Or_T1w_CE_Whole_RFE	DT	0.82857143	0.83333333	0.8	0.85714286	0.84166667
Or_T1w_CE_Whole_mRmR	ET	0.82857143	0.83333333	0.8	0.85714286	0.84166667
Or_T1w_CE_Whole_mRmR	GNB	0.82857143	0.83333333	0.8	0.85714286	0.84166667
Or_T1w_CE_Whole_mRmR	GB	0.82857143	0.83333333	0.8	0.85714286	0.84166667
Or_T1w_CE_Whole_mRmR	KN	0.82857143	0.83333333	0.8	0.85714286	0.84166667
Or_T1w_CE_Whole_mRmR	LDA	0.82857143	0.83333333	0.8	0.85714286	0.84166667
Or_T1w_CE_Whole_mRmR	MLPC	0.82857143	0.83333333	0.8	0.85714286	0.84166667
Or_T1w_CE_Whole_mRmR	NC	0.82857143	0.83333333	0.8	0.85714286	0.84166667
Or_T1w_CE_Whole_mRmR	PA	0.82857143	0.83333333	0.8	0.85714286	0.84166667
Or_T1w_CE_Whole_mRmR	QDA	0.82857143	0.83333333	0.8	0.85714286	0.84166667
Or_T1w_CE_Whole_mRmR	RF	0.82857143	0.83333333	0.8	0.85714286	0.84166667
Or_T1w_CE_Whole_mRmR	Ridge	0.82857143	0.83333333	0.8	0.85714286	0.84166667
Or_T1w_CE_Whole_mRmR	SVC	0.82857143	0.83333333	0.8	0.85714286	0.84166667
Or_T1w_CE_Whole_mRmR	DT	0.82857143	0.83333333	0.8	0.85714286	0.84166667

Or_T1w_CE_Whole_mRmR	ET	0.82857143	0.83333333	0.8	0.85714286	0.84166667
Or_T1w_CE_Whole_mRmR	QDA	0.82857143	0.83333333	0.8	0.85714286	0.84166667
Or_T1w_CE_Whole_mRmR	KN	0.8	0.83333333	0.6	1	0.88333333
Or_T1w_CE_Whole_mRmR	LDA	0.8	0.83333333	0.6	1	0.88333333
Or_T1w_CE_Whole_mRmR	Ridge	0.8	0.83333333	0.6	1	0.88333333
Or_T2w_Flair_Core_Kbest	KN	0.8	0.83333333	0.6	1	0.88333333
Or_T2w_Flair_Core_Kbest	LR	0.8	0.83333333	0.6	1	0.88333333
Or_T2w_Flair_Core_Kbest	MLPC	0.8	0.83333333	0.6	1	0.88333333
Or_T2w_Flair_Core_Kbest	ET	0.8	0.83333333	0.6	1	0.88333333
Or_T2w_Flair_Core_Kbest	GB	0.8	0.83333333	0.6	1	0.88333333
Or_T2w_Flair_Core_Kbest	MLPC	0.8	0.83333333	0.6	1	0.88333333
Or_T2w_Flair_Core_Kbest	RF	0.8	0.83333333	0.6	1	0.88333333
Or_T2w_Flair_Core_Kbest	Bagging	0.8	0.83333333	0.6	1	0.88333333
Or_T2w_Flair_Core_Kbest	ET	0.8	0.83333333	0.6	1	0.88333333
Or_T2w_Flair_Core_Kbest	GNB	0.8	0.83333333	0.6	1	0.88333333
Or_T2w_Flair_Core_Kbest	NC	0.8	0.83333333	0.6	1	0.88333333
Or_T2w_Flair_Core_Kbest	QDA	0.8	0.83333333	0.6	1	0.88333333
Or_T2w_Flair_Core_Kbest	RF	0.8	0.83333333	0.6	1	0.88333333
Or_T2w_Flair_Core_Kbest	Bagging	0.8	0.83333333	0.6	1	0.88333333
Or_T2w_Flair_Core_Kbest	ET	0.8	0.83333333	0.6	1	0.88333333
Or_T2w_Flair_Core_Kbest	GB	0.8	0.83333333	0.6	1	0.88333333
Or_T2w_Flair_Core_Kbest	KN	0.8	0.83333333	0.6	1	0.88333333
Or_T2w_Flair_Core_Kbest	NC	0.8	0.83333333	0.6	1	0.88333333
Or_T2w_Flair_Core_RFE	RF	0.8	0.83333333	0.6	1	0.88333333
Or_T2w_Flair_Core_RFE	Bagging	0.8	0.83333333	0.6	1	0.88333333
Or_T2w_Flair_Core_RFE	HGB	0.8	0.83333333	0.6	1	0.88333333
Or_T2w_Flair_Core_RFE	MLPC	0.8	0.83333333	0.6	1	0.88333333
Or_T2w_Flair_Core_RFE	Ridge	0.8	0.83333333	0.6	1	0.88333333
Or_T2w_Flair_Core_RFE	NC	0.8	0.83333333	0.6	1	0.88333333
Or_T2w_Flair_Core_RFE	RF	0.8	0.83333333	0.6	1	0.88333333
Or_T2w_Flair_Core_RFE	DT	0.8	0.83333333	0.6	1	0.88333333
Or_T2w_Flair_Core_RFE	LDA	0.8	0.83333333	0.6	1	0.88333333
Or_T2w_Flair_Core_RFE	AB	0.8	0.83333333	0.6	1	0.88333333
Or_T2w_Flair_Core_RFE	Bagging	0.8	0.83333333	0.6	1	0.88333333
Or_T2w_Flair_Core_RFE	GB	0.8	0.83333333	0.6	1	0.88333333
Or_T2w_Flair_Core_RFE	HGB	0.8	0.83333333	0.6	1	0.88333333
Or_T2w_Flair_Core_RFE	KN	0.8	0.83333333	0.6	1	0.88333333
Or_T2w_Flair_Core_RFE	MLPC	0.8	0.83333333	0.6	1	0.88333333
Or_T2w_Flair_Core_RFE	PA	0.8	0.83333333	0.6	1	0.88333333
Or_T2w_Flair_Core_RFE	SVC	0.8	0.83333333	0.6	1	0.88333333
Or_T2w_Flair_Core_RFE	GNB	0.78571429	0.75	1	0.57142857	0.8125
Or_T2w_Flair_Core_mRmR	GNB	0.78571429	0.75	1	0.57142857	0.8125
Or_T2w_Flair_Core_mRmR	HGB	0.78571429	0.75	1	0.57142857	0.8125
Or_T2w_Flair_Core_mRmR	HGB	0.78571429	0.75	1	0.57142857	0.8125

Or_T2w_Flair_Core_mRmR	SVC	0.78571429	0.75	1	0.57142857	0.8125
Or_T2w_Flair_Core_mRmR	DT	0.75714286	0.75	0.8	0.71428571	0.775
Or_T2w_Flair_Core_mRmR	GB	0.75714286	0.75	0.8	0.71428571	0.775
Or_T2w_Flair_Core_mRmR	Ridge	0.75714286	0.75	0.8	0.71428571	0.775
Or_T2w_Flair_Core_mRmR	DT	0.75714286	0.75	0.8	0.71428571	0.775
Or_T2w_Flair_Core_mRmR	KN	0.75714286	0.75	0.8	0.71428571	0.775
Or_T2w_Flair_Core_mRmR	HGB	0.75714286	0.75	0.8	0.71428571	0.775
Or_T2w_Flair_Core_mRmR	HGB	0.75714286	0.75	0.8	0.71428571	0.775
Or_T2w_Flair_Core_mRmR	GNB	0.75714286	0.75	0.8	0.71428571	0.775
Or_T2w_Flair_Core_mRmR	HGB	0.72857143	0.75	0.6	0.85714286	0.75833333
Or_T2w_Flair_Core_mRmR	HGB	0.72857143	0.75	0.6	0.85714286	0.75833333
Or_T2w_Flair_Core_mRmR	LR	0.72857143	0.75	0.6	0.85714286	0.75833333
Or_T2w_Flair_Core_mRmR	Ridge	0.72857143	0.75	0.6	0.85714286	0.75833333
Or_T2w_Flair_Core_mRmR	SVC	0.72857143	0.75	0.6	0.85714286	0.75833333
Or_T2w_Flair_Core_mRmR	AB	0.72857143	0.75	0.6	0.85714286	0.75833333
Or_T2w_Flair_Edema_Kbest	Bagging	0.72857143	0.75	0.6	0.85714286	0.75833333
Or_T2w_Flair_Edema_Kbest	DT	0.72857143	0.75	0.6	0.85714286	0.75833333
Or_T2w_Flair_Edema_Kbest	HGB	0.72857143	0.75	0.6	0.85714286	0.75833333
Or_T2w_Flair_Edema_Kbest	LR	0.72857143	0.75	0.6	0.85714286	0.75833333
Or_T2w_Flair_Edema_Kbest	PA	0.72857143	0.75	0.6	0.85714286	0.75833333
Or_T2w_Flair_Edema_Kbest	LR	0.72857143	0.75	0.6	0.85714286	0.75833333
Or_T2w_Flair_Edema_Kbest	AB	0.72857143	0.75	0.6	0.85714286	0.75833333
Or_T2w_Flair_Edema_Kbest	DT	0.72857143	0.75	0.6	0.85714286	0.75833333
Or_T2w_Flair_Edema_Kbest	HGB	0.72857143	0.75	0.6	0.85714286	0.75833333
Or_T2w_Flair_Edema_Kbest	LDA	0.72857143	0.75	0.6	0.85714286	0.75833333
Or_T2w_Flair_Edema_Kbest	QDA	0.72857143	0.75	0.6	0.85714286	0.75833333
Or_T2w_Flair_Edema_Kbest	Ridge	0.72857143	0.75	0.6	0.85714286	0.75833333
Or_T2w_Flair_Edema_Kbest	SVC	0.72857143	0.75	0.6	0.85714286	0.75833333
Or_T2w_Flair_Edema_Kbest	AB	0.72857143	0.75	0.6	0.85714286	0.75833333
Or_T2w_Flair_Edema_Kbest	GNB	0.72857143	0.75	0.6	0.85714286	0.75833333
Or_T2w_Flair_Edema_Kbest	KN	0.72857143	0.75	0.6	0.85714286	0.75833333
Or_T2w_Flair_Edema_Kbest	LDA	0.72857143	0.75	0.6	0.85714286	0.75833333
Or_T2w_Flair_Edema_Kbest	MLPC	0.72857143	0.75	0.6	0.85714286	0.75833333
Or_T2w_Flair_Edema_RFE	QDA	0.72857143	0.75	0.6	0.85714286	0.75833333
Or_T2w_Flair_Edema_RFE	HGB	0.72857143	0.75	0.6	0.85714286	0.75833333
Or_T2w_Flair_Edema_RFE	KN	0.72857143	0.75	0.6	0.85714286	0.75833333
Or_T2w_Flair_Edema_RFE	LDA	0.72857143	0.75	0.6	0.85714286	0.75833333
Or_T2w_Flair_Edema_RFE	MLPC	0.72857143	0.75	0.6	0.85714286	0.75833333
Or_T2w_Flair_Edema_RFE	PA	0.72857143	0.75	0.6	0.85714286	0.75833333
Or_T2w_Flair_Edema_RFE	QDA	0.72857143	0.75	0.6	0.85714286	0.75833333
Or_T2w_Flair_Edema_RFE	Ridge	0.72857143	0.75	0.6	0.85714286	0.75833333
Or_T2w_Flair_Edema_RFE	SVC	0.72857143	0.75	0.6	0.85714286	0.75833333
Or_T2w_Flair_Edema_RFE	LR	0.72857143	0.75	0.6	0.85714286	0.75833333
Or_T2w_Flair_Edema_RFE	LR	0.72857143	0.75	0.6	0.85714286	0.75833333

Or_T2w_Flair_Edema_RFE	AB	0.72857143	0.75	0.6	0.85714286	0.75833333
Or_T2w_Flair_Edema_RFE	KN	0.72857143	0.75	0.6	0.85714286	0.75833333
Or_T2w_Flair_Edema_RFE	NC	0.72857143	0.75	0.6	0.85714286	0.75833333
Or_T2w_Flair_Edema_RFE	QDA	0.72857143	0.75	0.6	0.85714286	0.75833333
Or_T2w_Flair_Edema_RFE	SVC	0.72857143	0.75	0.6	0.85714286	0.75833333
Or_T2w_Flair_Edema_RFE	NC	0.71428571	0.66666667	1	0.42857143	0.77777778
Or_T2w_Flair_Edema_RFE	NC	0.71428571	0.66666667	1	0.42857143	0.77777778
Or_T2w_Flair_Edema_mRmR	HGB	0.71428571	0.66666667	1	0.42857143	0.77777778
Or_T2w_Flair_Edema_mRmR	NC	0.71428571	0.66666667	1	0.42857143	0.77777778
Or_T2w_Flair_Edema_mRmR	LDA	0.7	0.75	0.4	1	0.825
Or_T2w_Flair_Edema_mRmR	LR	0.7	0.75	0.4	1	0.825
Or_T2w_Flair_Edema_mRmR	LR	0.7	0.75	0.4	1	0.825
Or_T2w_Flair_Edema_mRmR	LR	0.7	0.75	0.4	1	0.825
Or_T2w_Flair_Edema_mRmR	LR	0.7	0.75	0.4	1	0.825
Or_T2w_Flair_Edema_mRmR	AB	0.7	0.75	0.4	1	0.825
Or_T2w_Flair_Edema_mRmR	LR	0.7	0.75	0.4	1	0.825
Or_T2w_Flair_Edema_mRmR	PA	0.7	0.75	0.4	1	0.825
Or_T2w_Flair_Edema_mRmR	LDA	0.7	0.75	0.4	1	0.825
Or_T2w_Flair_Edema_mRmR	LDA	0.7	0.75	0.4	1	0.825
Or_T2w_Flair_Edema_mRmR	LR	0.7	0.75	0.4	1	0.825
Or_T2w_Flair_Edema_mRmR	Ridge	0.7	0.75	0.4	1	0.825
Or_T2w_Flair_Edema_mRmR	BNB	0.7	0.75	0.4	1	0.825
Or_T2w_Flair_Edema_mRmR	KN	0.7	0.75	0.4	1	0.825
Or_T2w_Flair_Edema_mRmR	LDA	0.7	0.75	0.4	1	0.825
Or_T2w_Flair_Edema_mRmR	MLPC	0.7	0.75	0.4	1	0.825
Or_T2w_Flair_Enhancing_Kbest	PA	0.7	0.75	0.4	1	0.825
Or_T2w_Flair_Enhancing_Kbest	Ridge	0.7	0.75	0.4	1	0.825
Or_T2w_Flair_Enhancing_Kbest	SVC	0.7	0.75	0.4	1	0.825
Or_T2w_Flair_Enhancing_Kbest	Ridge	0.7	0.75	0.4	1	0.825
Or_T2w_Flair_Enhancing_Kbest	PA	0.7	0.75	0.4	1	0.825
Or_T2w_Flair_Enhancing_Kbest	HGB	0.7	0.75	0.4	1	0.825
Or_T2w_Flair_Enhancing_Kbest	GB	0.7	0.75	0.4	1	0.825
Or_T2w_Flair_Enhancing_Kbest	PA	0.7	0.75	0.4	1	0.825
Or_T2w_Flair_Enhancing_Kbest	RF	0.7	0.75	0.4	1	0.825
Or_T2w_Flair_Enhancing_Kbest	GNB	0.7	0.75	0.4	1	0.825
Or_T2w_Flair_Enhancing_Kbest	QDA	0.7	0.75	0.4	1	0.825
Or_T2w_Flair_Enhancing_Kbest	ET	0.7	0.75	0.4	1	0.825
Or_T2w_Flair_Enhancing_Kbest	GB	0.7	0.75	0.4	1	0.825
Or_T2w_Flair_Enhancing_Kbest	HGB	0.7	0.75	0.4	1	0.825
Or_T2w_Flair_Enhancing_Kbest	LR	0.7	0.75	0.4	1	0.825
Or_T2w_Flair_Enhancing_Kbest	NC	0.7	0.75	0.4	1	0.825
Or_T2w_Flair_Enhancing_Kbest	RF	0.7	0.75	0.4	1	0.825
Or_T2w_Flair_Enhancing_Kbest	Ridge	0.7	0.75	0.4	1	0.825
Or_T2w_Flair_Enhancing_RFE	LDA	0.7	0.75	0.4	1	0.825

Or_T2w_Flair_Enhancing_RFE	LR	0.7	0.75	0.4	1	0.825
Or_T2w_Flair_Enhancing_RFE	Ridge	0.7	0.75	0.4	1	0.825
Or_T2w_Flair_Enhancing_RFE	HGB	0.68571429	0.66666667	0.8	0.57142857	0.72738095
Or_T2w_Flair_Enhancing_RFE	QDA	0.68571429	0.66666667	0.8	0.57142857	0.72738095
Or_T2w_Flair_Enhancing_RFE	QDA	0.68571429	0.66666667	0.8	0.57142857	0.72738095
Or_T2w_Flair_Enhancing_RFE	NC	0.68571429	0.66666667	0.8	0.57142857	0.72738095
Or_T2w_Flair_Enhancing_RFE	HGB	0.68571429	0.66666667	0.8	0.57142857	0.72738095
Or_T2w_Flair_Enhancing_RFE	HGB	0.68571429	0.66666667	0.8	0.57142857	0.72738095
Or_T2w_Flair_Enhancing_RFE	HGB	0.68571429	0.66666667	0.8	0.57142857	0.72738095
Or_T2w_Flair_Enhancing_RFE	QDA	0.68571429	0.66666667	0.8	0.57142857	0.72738095
Or_T2w_Flair_Enhancing_RFE	AB	0.65714286	0.66666667	0.6	0.71428571	0.68333333
Or_T2w_Flair_Enhancing_RFE	Bagging	0.65714286	0.66666667	0.6	0.71428571	0.68333333
Or_T2w_Flair_Enhancing_RFE	DT	0.65714286	0.66666667	0.6	0.71428571	0.68333333
Or_T2w_Flair_Enhancing_RFE	ET	0.65714286	0.66666667	0.6	0.71428571	0.68333333
Or_T2w_Flair_Enhancing_RFE	GNB	0.65714286	0.66666667	0.6	0.71428571	0.68333333
Or_T2w_Flair_Enhancing_RFE	GB	0.65714286	0.66666667	0.6	0.71428571	0.68333333
Or_T2w_Flair_Enhancing_RFE	KN	0.65714286	0.66666667	0.6	0.71428571	0.68333333
Or_T2w_Flair_Enhancing_mRmR	MLPC	0.65714286	0.66666667	0.6	0.71428571	0.68333333
Or_T2w_Flair_Enhancing_mRmR	PA	0.65714286	0.66666667	0.6	0.71428571	0.68333333
Or_T2w_Flair_Enhancing_mRmR	RF	0.65714286	0.66666667	0.6	0.71428571	0.68333333
Or_T2w_Flair_Enhancing_mRmR	ET	0.65714286	0.66666667	0.6	0.71428571	0.68333333
Or_T2w_Flair_Enhancing_mRmR	GNB	0.65714286	0.66666667	0.6	0.71428571	0.68333333
Or_T2w_Flair_Enhancing_mRmR	LR	0.65714286	0.66666667	0.6	0.71428571	0.68333333
Or_T2w_Flair_Enhancing_mRmR	ET	0.65714286	0.66666667	0.6	0.71428571	0.68333333
Or_T2w_Flair_Enhancing_mRmR	GNB	0.65714286	0.66666667	0.6	0.71428571	0.68333333
Or_T2w_Flair_Enhancing_mRmR	LR	0.65714286	0.66666667	0.6	0.71428571	0.68333333
Or_T2w_Flair_Enhancing_mRmR	RF	0.65714286	0.66666667	0.6	0.71428571	0.68333333
Or_T2w_Flair_Enhancing_mRmR	Ridge	0.65714286	0.66666667	0.6	0.71428571	0.68333333
Or_T2w_Flair_Enhancing_mRmR	SVC	0.65714286	0.66666667	0.6	0.71428571	0.68333333
Or_T2w_Flair_Enhancing_mRmR	AB	0.65714286	0.66666667	0.6	0.71428571	0.68333333
Or_T2w_Flair_Enhancing_mRmR	ET	0.65714286	0.66666667	0.6	0.71428571	0.68333333
Or_T2w_Flair_Enhancing_mRmR	HGB	0.65714286	0.66666667	0.6	0.71428571	0.68333333
Or_T2w_Flair_Enhancing_mRmR	RF	0.65714286	0.66666667	0.6	0.71428571	0.68333333
Or_T2w_Flair_Enhancing_mRmR	GNB	0.65714286	0.66666667	0.6	0.71428571	0.68333333
Or_T2w_Flair_Enhancing_mRmR	HGB	0.64285714	0.58333333	1	0.28571429	0.75
Or_T2w_Flair_Necrosis_Kbest	PA	0.64285714	0.58333333	1	0.28571429	0.75
Or_T2w_Flair_Enhancing_Kbest	LDA	0.62857143	0.66666667	0.4	0.85714286	0.65833333
Or_T2w_Flair_Enhancing_Kbest	DT	0.62857143	0.66666667	0.4	0.85714286	0.65833333
Or_T2w_Flair_Enhancing_Kbest	GNB	0.62857143	0.66666667	0.4	0.85714286	0.65833333
Or_T2w_Flair_Enhancing_Kbest	QDA	0.62857143	0.66666667	0.4	0.85714286	0.65833333
Or_T2w_Flair_Enhancing_Kbest	SVC	0.62857143	0.66666667	0.4	0.85714286	0.65833333
Or_T2w_Flair_Enhancing_Kbest	AB	0.62857143	0.66666667	0.4	0.85714286	0.65833333
Or_T2w_Flair_Enhancing_Kbest	KN	0.62857143	0.66666667	0.4	0.85714286	0.65833333
Or_T2w_Flair_Enhancing_Kbest	RF	0.62857143	0.66666667	0.4	0.85714286	0.65833333

Or_T2w_Flair_Enhancing_Kbest	KN	0.62857143	0.66666667	0.4	0.85714286	0.65833333
Or_T2w_Flair_Enhancing_Kbest	LR	0.62857143	0.66666667	0.4	0.85714286	0.65833333
Or_T2w_Flair_Enhancing_Kbest	MLPC	0.62857143	0.66666667	0.4	0.85714286	0.65833333
Or_T2w_Flair_Enhancing_Kbest	PA	0.62857143	0.66666667	0.4	0.85714286	0.65833333
Or_T2w_Flair_Enhancing_Kbest	Ridge	0.62857143	0.66666667	0.4	0.85714286	0.65833333
Or_T2w_Flair_Enhancing_Kbest	LR	0.62857143	0.66666667	0.4	0.85714286	0.65833333
Or_T2w_Flair_Enhancing_Kbest	AB	0.62857143	0.66666667	0.4	0.85714286	0.65833333
Or_T2w_Flair_Enhancing_Kbest	Bagging	0.62857143	0.66666667	0.4	0.85714286	0.65833333
Or_T2w_Flair_Enhancing_Kbest	KN	0.62857143	0.66666667	0.4	0.85714286	0.65833333
Or_T2w_Flair_Enhancing_RFE	MLPC	0.62857143	0.66666667	0.4	0.85714286	0.65833333
Or_T2w_Flair_Enhancing_RFE	PA	0.62857143	0.66666667	0.4	0.85714286	0.65833333
Or_T2w_Flair_Enhancing_RFE	SVC	0.62857143	0.66666667	0.4	0.85714286	0.65833333
Or_T2w_Flair_Enhancing_RFE	GNB	0.62857143	0.66666667	0.4	0.85714286	0.65833333
Or_T2w_Flair_Enhancing_RFE	GB	0.62857143	0.66666667	0.4	0.85714286	0.65833333
Or_T2w_Flair_Enhancing_RFE	LR	0.62857143	0.66666667	0.4	0.85714286	0.65833333
Or_T2w_Flair_Enhancing_RFE	MLPC	0.62857143	0.66666667	0.4	0.85714286	0.65833333
Or_T2w_Flair_Enhancing_RFE	Ridge	0.62857143	0.66666667	0.4	0.85714286	0.65833333
Or_T2w_Flair_Enhancing_RFE	NC	0.62857143	0.66666667	0.4	0.85714286	0.65833333
Or_T2w_Flair_Enhancing_RFE	AB	0.61428571	0.58333333	0.8	0.42857143	0.69166667
Or_T2w_Flair_Enhancing_RFE	Bagging	0.61428571	0.58333333	0.8	0.42857143	0.69166667
Or_T2w_Flair_Enhancing_RFE	DT	0.61428571	0.58333333	0.8	0.42857143	0.69166667
Or_T2w_Flair_Enhancing_RFE	Bagging	0.6	0.66666667	0.2	1	0.76666667
Or_T2w_Flair_Enhancing_RFE	GB	0.6	0.66666667	0.2	1	0.76666667
Or_T2w_Flair_Enhancing_RFE	BNB	0.6	0.66666667	0.2	1	0.76666667
Or_T2w_Flair_Enhancing_RFE	BNB	0.6	0.66666667	0.2	1	0.76666667
Or_T2w_Flair_Enhancing_RFE	LR	0.6	0.66666667	0.2	1	0.76666667
Or_T2w_Flair_Enhancing_RFE	HGB	0.6	0.66666667	0.2	1	0.76666667
Or_T2w_Flair_Enhancing_mRmR	ET	0.6	0.66666667	0.2	1	0.76666667
Or_T2w_Flair_Enhancing_mRmR	LDA	0.6	0.66666667	0.2	1	0.76666667
Or_T2w_Flair_Enhancing_mRmR	LR	0.6	0.66666667	0.2	1	0.76666667
Or_T2w_Flair_Enhancing_mRmR	MLPC	0.6	0.66666667	0.2	1	0.76666667
Or_T2w_Flair_Enhancing_mRmR	Ridge	0.6	0.66666667	0.2	1	0.76666667
Or_T2w_Flair_Enhancing_mRmR	SVC	0.6	0.66666667	0.2	1	0.76666667
Or_T2w_Flair_Enhancing_mRmR	BNB	0.6	0.66666667	0.2	1	0.76666667
Or_T2w_Flair_Enhancing_mRmR	BNB	0.6	0.66666667	0.2	1	0.76666667
Or_T2w_Flair_Enhancing_mRmR	BNB	0.6	0.66666667	0.2	1	0.76666667
Or_T2w_Flair_Enhancing_mRmR	NC	0.58571429	0.58333333	0.6	0.57142857	0.63333333
Or_T2w_Flair_Enhancing_mRmR	KN	0.58571429	0.58333333	0.6	0.57142857	0.63333333
Or_T2w_Flair_Enhancing_mRmR	Bagging	0.58571429	0.58333333	0.6	0.57142857	0.63333333
Or_T2w_Flair_Enhancing_mRmR	MLPC	0.58571429	0.58333333	0.6	0.57142857	0.63333333
Or_T2w_Flair_Enhancing_mRmR	NC	0.58571429	0.58333333	0.6	0.57142857	0.63333333
Or_T2w_Flair_Enhancing_mRmR	AB	0.58571429	0.58333333	0.6	0.57142857	0.63333333
Or_T2w_Flair_Enhancing_mRmR	GNB	0.58571429	0.58333333	0.6	0.57142857	0.63333333
Or_T2w_Flair_Enhancing_mRmR	PA	0.58571429	0.58333333	0.6	0.57142857	0.63333333

Or_T2w_Flair_Enhancing_mRmR	LDA	0.55714286	0.58333333	0.4	0.71428571	0.575
Or_T2w_Flair_Whole_Kbest	Bagging	0.55714286	0.58333333	0.4	0.71428571	0.575
Or_T2w_Flair_Whole_Kbest	GB	0.55714286	0.58333333	0.4	0.71428571	0.575
Or_T2w_Flair_Whole_Kbest	QDA	0.52857143	0.58333333	0.2	0.85714286	0.51666667
Or_T2w_Flair_Whole_Kbest	DT	0.52857143	0.58333333	0.2	0.85714286	0.51666667
Or_T2w_Flair_Whole_Kbest	LDA	0.52857143	0.58333333	0.2	0.85714286	0.51666667
Or_T2w_Flair_Whole_Kbest	NC	0.52857143	0.58333333	0.2	0.85714286	0.51666667
Or_T2w_Flair_Whole_Kbest	QDA	0.52857143	0.58333333	0.2	0.85714286	0.51666667
Or_T2w_Flair_Whole_Kbest	ET	0.52857143	0.58333333	0.2	0.85714286	0.51666667
Or_T2w_Flair_Whole_Kbest	KN	0.52857143	0.58333333	0.2	0.85714286	0.51666667
Or_T2w_Flair_Whole_Kbest	NC	0.52857143	0.58333333	0.2	0.85714286	0.51666667
Or_T2w_Flair_Whole_Kbest	BNB	0.52857143	0.58333333	0.2	0.85714286	0.51666667
Or_T2w_Flair_Whole_Kbest	RF	0.51428571	0.5	0.6	0.42857143	0.59761905
Or_T2w_Flair_Whole_Kbest	AB	0.51428571	0.5	0.6	0.42857143	0.59761905
Or_T2w_Flair_Whole_Kbest	BNB	0.5	0.58333333	0	1	0.70833333
Or_T2w_Flair_Whole_Kbest	BNB	0.5	0.58333333	0	1	0.70833333
Or_T2w_Flair_Whole_Kbest	BNB	0.5	0.58333333	0	1	0.70833333
Or_T2w_Flair_Whole_Kbest	QDA	0.5	0.58333333	0	1	0.70833333
Or_T2w_Flair_Whole_Kbest	BNB	0.5	0.58333333	0	1	0.70833333
Or_T2w_Flair_Whole_RFE	BNB	0.5	0.58333333	0	1	0.70833333
Or_T2w_Flair_Whole_RFE	BNB	0.5	0.58333333	0	1	0.70833333
Or_T2w_Flair_Whole_RFE	BNB	0.5	0.58333333	0	1	0.70833333
Or_T2w_Flair_Whole_RFE	BNB	0.5	0.58333333	0	1	0.70833333
Or_T2w_Flair_Whole_RFE	BNB	0.5	0.58333333	0	1	0.70833333
Or_T2w_Flair_Whole_RFE	BNB	0.5	0.58333333	0	1	0.70833333
Or_T2w_Flair_Whole_RFE	BNB	0.5	0.58333333	0	1	0.70833333
Or_T2w_Flair_Whole_RFE	BNB	0.5	0.58333333	0	1	0.70833333
Or_T2w_Flair_Whole_RFE	LR	0.5	0.58333333	0	1	0.70833333
Or_T2w_Flair_Whole_RFE	BNB	0.5	0.58333333	0	1	0.70833333
Or_T2w_Flair_Whole_RFE	BNB	0.5	0.58333333	0	1	0.70833333
Or_T2w_Flair_Whole_RFE	BNB	0.5	0.58333333	0	1	0.70833333
Or_T2w_Flair_Whole_RFE	KN	0.5	0.58333333	0	1	0.70833333
Or_T2w_Flair_Whole_RFE	LR	0.5	0.58333333	0	1	0.70833333
Or_T2w_Flair_Whole_RFE	MLPC	0.5	0.58333333	0	1	0.70833333
Or_T2w_Flair_Whole_RFE	Ridge	0.5	0.58333333	0	1	0.70833333
Or_T2w_Flair_Whole_RFE	SVC	0.5	0.58333333	0	1	0.70833333
Or_T2w_Flair_Whole_RFE	LR	0.5	0.58333333	0	1	0.70833333
Or_T2w_Flair_Whole_mRmR	BNB	0.5	0.58333333	0	1	0.70833333
Or_T2w_Flair_Whole_mRmR	BNB	0.5	0.58333333	0	1	0.70833333
Or_T2w_Flair_Whole_mRmR	BNB	0.5	0.58333333	0	1	0.70833333
Or_T2w_Flair_Whole_mRmR	BNB	0.5	0.58333333	0	1	0.70833333
Or_T2w_Flair_Whole_mRmR	BNB	0.5	0.58333333	0	1	0.70833333
Or_T2w_Flair_Whole_mRmR	BNB	0.5	0.58333333	0	1	0.70833333
Or_T2w_Flair_Whole_mRmR	AB	0.45714286	0.5	0.2	0.71428571	0.43333333

Or_T2w_Flair_Whole_mRmR	ET	0.45714286	0.5	0.2	0.71428571	0.43333333
Or_T2w_Flair_Whole_mRmR	RF	0.45714286	0.5	0.2	0.71428571	0.43333333
Or_T2w_Flair_Whole_mRmR	BNB	0.42857143	0.5	0	0.85714286	0.20833333
Or_T2w_Flair_Whole_mRmR	Bagging	0.42857143	0.5	0	0.85714286	0.20833333
Or_T2w_Flair_Whole_mRmR	GNB	0.42857143	0.5	0	0.85714286	0.20833333
Or_T2w_Flair_Whole_mRmR	GB	0.42857143	0.5	0	0.85714286	0.20833333
Or_T2w_Flair_Whole_mRmR	PA	0.42857143	0.5	0	0.85714286	0.20833333
Or_T2w_Flair_Whole_mRmR	PA	0.42857143	0.5	0	0.85714286	0.20833333
Or_T2w_Flair_Whole_mRmR	LR	0.42857143	0.5	0	0.85714286	0.20833333
Or_T2w_Flair_Whole_mRmR	GB	0.41428571	0.41666667	0.4	0.42857143	0.49166667
Or_T2w_Flair_Whole_mRmR	BNB	0.38571429	0.41666667	0.2	0.57142857	0.39166667
